# A photocontrolled one-pot isothermal amplification and CRISPR-Cas12a assay for rapid detection of SARS-CoV-2 Omicron variants

**DOI:** 10.1128/spectrum.03645-23

**Published:** 2024-02-06

**Authors:** Qian Sun, Hongqing Lin, Yuan Li, Liping Yuan, Baisheng Li, Yunan Ma, Haiying Wang, Xiaoling Deng, Hongliang Chen, Shixing Tang

**Affiliations:** 1 Guangdong Provincial Key Laboratory of Tropical Disease Research, Department of Epidemiology, School of Public Health, Southern Medical University, Guangzhou, Guangdong, China; 2 Institute of Pathogenic Microbiology, Guangdong Provincial Center for Disease Control and Prevention, Guangdong Workstation for Emerging Infectious Disease Control and Prevention, Chinese Academy of Medical Sciences, Guangzhou, China; 3 Department of Clinical Microbiology Laboratory, Chenzhou No. 1 People’s Hospital, Chenzhou, China; 4 Department of Infectious Diseases, Nanfang Hospital, Southern Medical University, Guangzhou, China; Karolinska Institutet, Stockholm, Sweden

**Keywords:** CRISPR-Cas12a, photocleavage, isothermal amplification, one-pot assay, SARS-CoV-2, Omicron variant, genotyping

## Abstract

CRISPR-Cas technology has widely been applied to detect single-nucleotide mutation and is considered as the next generation of molecular diagnostics. We previously reported the combination of nucleic acid amplification (NAA) and CRISPR-Cas12a system to distinguish major severe acute respiratory syndrome coronavirus 2 (SARS-CoV-2) variants. However, the mixture of NAA and CRISPR-Cas12a reagents in one tube could interfere with the efficiency of NAA and CRISPR-Cas12a cleavage, which in turn affects the detection sensitivity. In the current study, we employed a novel photoactivated CRISPR-Cas12a strategy integrated with recombinase polymerase amplification (RPA) to develop one-pot RPA/CRISPR-Cas12a genotyping assay for detecting SARS-CoV-2 Omicron sub-lineages. The new system overcomes the potential inhibition of RPA due to early CRISPR-Cas12a activation and cleavage of the target template in traditional one-pot assay using photocleavable p-RNA, a complementary single-stranded RNA to specifically bind crRNA and precisely block Cas12a activation. The detection can be finished in one tube at 39℃ within 1 h and exhibits a low limit of detection of 30 copies per reaction. Our results demonstrated that the photocontrolled one-pot RPA/CRISPR-Cas12a assay could effectively identify three signature mutations in the spike gene of SARS-CoV-2 Omicron variant, namely, R346T, F486V, and 49X, and distinguish Omicron BA.1, BA.5.2, and BF.7 sub-lineages. Furthermore, the assay achieved a sensitivity of 97.3% and a specificity of 100.0% and showed a concordance of 98.3% with Sanger sequencing results.

**IMPORTANCE:**

We successfully developed one-pot recombinase polymerase amplification/CRISPR-Cas12a genotyping assay by adapting photocontrolled CRISPR-Cas technology to optimize the conditions of nucleic acid amplification and CRISPR-Cas12a-mediated detection. This innovative approach was able to quickly distinguish severe acute respiratory syndrome coronavirus 2 Omicron variants and can be readily modified for detecting any nucleic acid mutations. The assay system demonstrates excellent clinical performance, including rapid detection, user-friendly operations, and minimized risk of contamination, which highlights its promising potential as a point-of-care testing for wide applications in resource-limiting settings.

## INTRODUCTION

The prokaryote-originated CRISPR-Cas system is critical in adaptive immunity by specifically recognizing and destroying foreign nucleic acids and is initially used as a gene-editing tool (1, 2). During the coronavirus disease 2019 (COVID-19) pandemic, the CRISPR system has also been adapted for detecting and genotyping severe acute respiratory syndrome coronavirus 2 (SARS-CoV-2) variants (3–5). It is considered the next generation of molecular diagnostics and may be a potential alternative to PCR or next-generation sequencing due to its rapid detection, simplicity, and high sensitivity and specificity (6, 7). In general, CRISPR-based assays often combine with nucleic acid amplification (NAA) techniques, such as PCR, loop-mediated isothermal amplification, or recombinase polymerase amplification (RPA), to increase detection sensitivity (8–10). However, NAA and CRISPR detection are usually carried out separately in two tubes, which in turn makes the operation time consuming and increases cross-contamination risk due to amplification product transfer. Therefore, different one-pot assay systems have been designed by integrating NAA and CRISPR reactions but do not affect the detection efficiency caused by early Cas activation and degradation of amplification products. The strategies to develop one-pot assay include (i) physical separation of the NAA and CRISPR system followed by moving the CRISPR system into the NAA mixture (11, 12), delayed fusion of the two phases of reagents by leveraging glycerol viscosity (13), or dynamic diffusion of the two reactions according to biphase density difference of sucrose concentration (14); (ii) inhibition of Cas activity using crRNA to recognize suboptimal protospacer adjacent motif (PAM) (15) or dual crRNAs without PAM limitation (16) to make the initial stage of reaction dominated by NAA. However, these solutions always lead to extra manual operations, complex design, increased costs, or poor feasibility.

Photocontrolled technique has been proven as a crucial tool for precise manipulation and regulation of CRISPR/Cas9-mediated gene editing (17, 18) and one-pot CRISPR detection (19–21) using a p-RNA, which is complementary to crRNA but contains photocleavable linkers (PC-linker) that can be destroyed through transient UV irradiation. The binding of p-RNA and crRNA blocks the binding of the crRNA with the target template, which in turn inhibits the activation of Cas enzyme and the cleavage of the target nucleic acid templates in one-pot reaction system. After sufficient amplification of the target nucleic acid templates, a brief irradiation of the reaction tube with 365 nm UV light results in the cleavage of p-RNA and dissociation of the p-RNA/crRNA complex (p-crRNA) to release crRNA. Consequently, free crRNA can bind to the target nucleic acid to activate Cas enzyme and initiate the CRISPR-Cas detection system.

**Fig 1:**
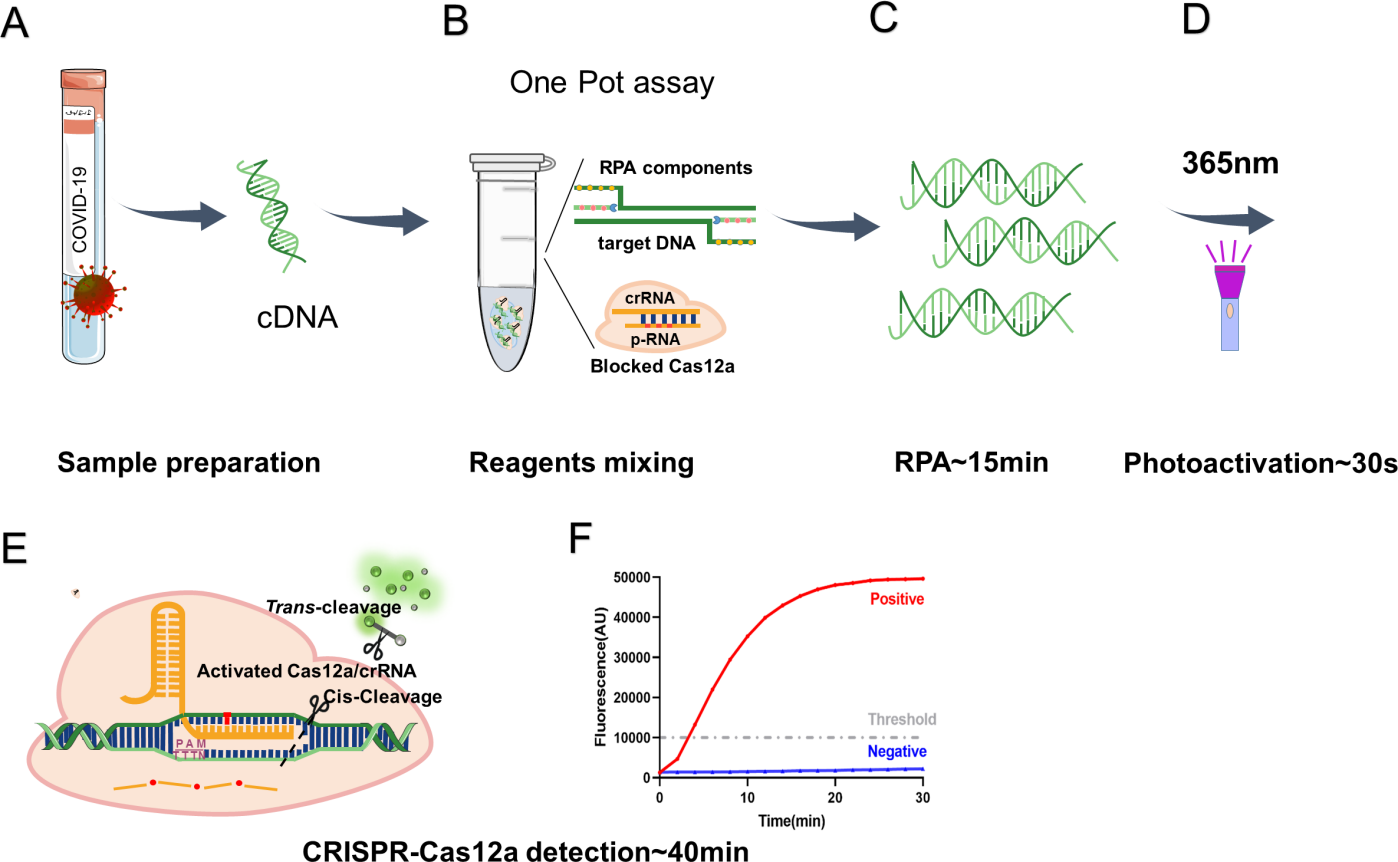
Workflow of the photocontrolled one-pot RPA/CRISPR-Cas12a assay. Briefly, viral RNA was extracted from SARS-CoV-2-positive serum or plasma samples and reverse transcribed into cDNA (**A**), which was transferred into one tube and mixed with RPA and CRISPR-Cas12a reagents (**B**). SARS-CoV-2 S gene was first amplified by RPA (**C**) followed by cleavage of p-RNA through UV light-induced photolysis to release crRNA (**D**). In the CRISPR-Cas12a assay, Cas12a was activated and cut the reporter to release fluorescence (**E**), which was recorded in real time by a fluorescence meter (**F**). All the detection was proceeded sequentially in one tube. p-RNA contains three photocleavable linkers (represented by red dots) and can specifically bind the corresponding crRNA based on base match.

CRISPR-based assays have been successfully used to distinguish SARS-CoV-2 variants (22–25). Our group has developed the CRISPR-Cas12a system by integrating PCR or recombinase-aided amplification (RAA) to detect major SARS-CoV-2 variants of concern and has demonstrated appreciable clinical performance (26, 27); however, these approaches are not a one-pot detection system. In the current study, we established a one-pot RPA/CRISPR-Cas12a platform by adapting the aforementioned photoactivatable crRNA strategy to precisely control the time of Cas12a activation and CRISPR detection to distinguish major SARS-CoV-2 Omicron sub-lineages including BA.1, BA.5.2, and BF.7 (Fig. 1).

## MATERIALS AND METHODS

### Clinical samples and reagents

A total of 57 SARS-CoV-2-positive pharyngeal swab specimens were included in this study. Among them, 45 samples were obtained from Guangdong Provincial Center for Disease Control and Prevention between March 2020 and June 2022, while the remaining 12 samples were obtained from Chenzhou First People’s Hospital in December 2022. Viral RNA was extracted from pharyngeal swab specimens and subsequently reverse transcribed into cDNA for further analysis. To evaluate the assay specificity, 11 SARS-CoV-2-negative clinical samples were collected prior to the COVID-19 pandemic and infected with various respiratory pathogens including common human coronaviruses 229E, OC43, and HKU1, as well as rhinovirus (HRV), adenovirus (ADV), respiratory syncytial virus (RSV) A and B, human bocavirus (HBoV), human metapneumovirus (HMPV), and human parainfluenza virus one (HPIV-1) and 4 (HPIV-4) (27). All the subjects involved in this study provided written informed consent, and ethical standards were maintained throughout the study.

Cas12a protein from *Acidaminococcus* sp. (AsCas12a) was expressed and purified in our laboratory (26). NEBuffer 2.1 (10×), buffer3.0 (10×), HiScribe T7 High Yield RNA Synthesis Kit, and Monarch RNA Cleanup Kit used for crRNA preparation were purchased from New England Biolabs (NEB, MA, USA). The Universal DNA Purification Kit was purchased from TIANGEN Biotech Co. (Beijing, China). The ssDNA reporter (5′−6-FAM-TTATT-BHQ-1–3′) and all the plasmids used in this study were synthesized in Sangon Biotech (Shanghai, China), and their sequences are provided in Table S1. The RPA and PCR primers as well as crRNAs were synthesized by Ruiboxingke Biotechnology (Beijing, China), and their details can be found in Table S2. The p-RNAs modified with PC linkers were synthesized by Bio-LifeSci (Guangzhou, China), and their sequences are listed in Table S3. ApexHF HS DNA Polymerase FS Mix and DNase/RNase-free water were obtained from Accurate Biotechnology (Hunan, China). RAA basic kit was obtained from Qitian Biotechnology (Jiangsu, China). TwistAmp Liquid Basic kit and TwistAmp Basic kit were obtained from TwistDx Limited. QIAamp Viral RNA Mini Kit used for SARS-COV-2 RNA extraction was purchased from Qiagen (Hilden, Germany), and Transcriptor First Strand cDNA Synthesis Kit was purchased from Roche Diagnostics (Indianapolis, USA). Chemical reagents, including dithiothreitol (DTT) and PEG-20000, were purchased from Sigma-Aldrich (St. Louis, Missouri, USA).

### Signature mutations specific for SARS-COV-2 Omicron BA.1, BA.5.2, and BF.7

The spike gene fragments of major SARS-CoV-2 Omicron sub-lineages from various geographic locations were downloaded from the National Center for Biotechnology Information Virus database and aligned with the wild-type SARS-CoV-2 strain (GenBank accession no. MN908947) using MAFFT version 7. The alignment results (Fig. S2) were visualized by ESPript 3.0 and revealed that three signature mutations in the spike protein, i.e., R346T, F486V, and 49X (covering Q493R, G496S, and Q498R), could distinguish Omicron sub-lineages BA.1, BA.5.2, and BF.7.

### Design and synthesis of crRNAs

**Fig 2:**
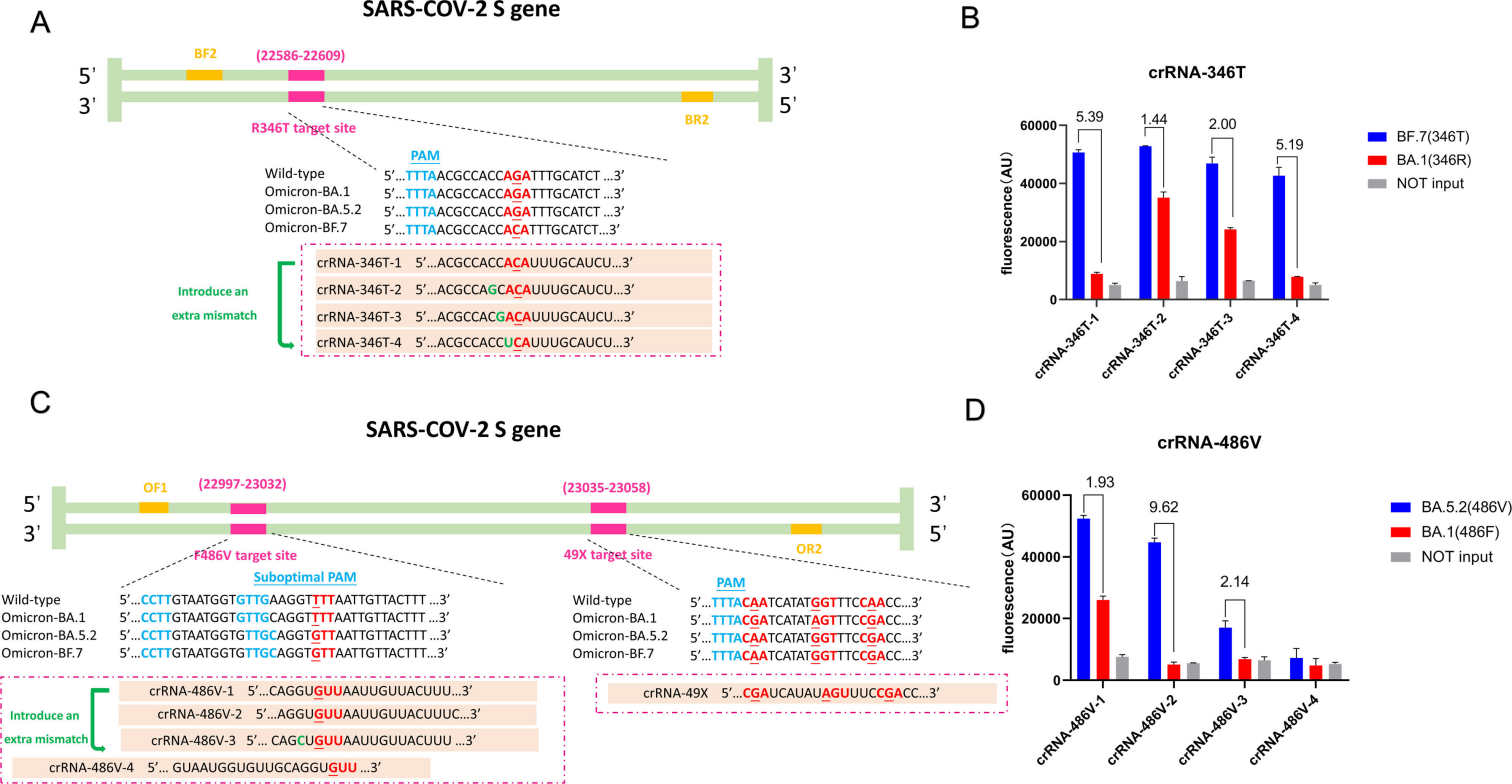
Design and selection of the crRNAs. The schematic and sequences of the SARS-CoV-2 spike gene with R346T mutation (**A**) or F486V and 49X mutations (**C**). PAM or suboptimal PAM is labeled in blue, RPA primers in yellow, and the crRNAs targeting the specific mutations in red. The performance of the crRNAs was evaluated in RPA/CRISPR-Cas12a assay using plasmid template of SARS-COV-2 Omicron (10^6^ copies/μL) with R346T (**B**) or F486V mutation (**D**). The endpoint fluorescence signal was measured and presented as positive in blue for Omicron BF.7 with 346T or BA.5.2 with 486V and negative in red for Omicron BA.1 with 346R or BA.1 with 486F. The number represents the ratio of positive over negative samples. No input means blank control and is labeled in gray.

crRNAs were designed to target the aforementioned three signature mutations and recognize PAM motif 5′-TTTV-3′, where V refers to A/G/C. For the S gene with mutations R346T and 49X, a PAM motif (5′-TTTA-3′) was found near the 5′ end of the target sequence. However, for the mutation F486V, no canonical PAM exists, and a suboptimal PAM (VTTV, TCTV, and TTVV) was used. The positions and sequences of the crRNAs were schematically depicted in Fig. 2A and C and can be found in Table S2.

To prepare the crRNAs, the corresponding DNA templates containing the T7 promoter and the complementary sequences were denatured in 1 × buffer3.0 at 95°C for 10 min, followed by gradually annealing to room temperature at a decline rate of 2°C/min. Subsequently, 1 µg of the purified dsDNA was transcribed at 37°C for 4 h, and the excess DNA template in the product was treated with 4 U of DNase I at 37°C for 40 min. The *in vitro* transcription process was performed using the HiScribe T7 High Yield RNA Synthesis Kit, and crRNA purification was carried out using the Monarch RNA Cleanup Kit. The concentration of crRNAs was determined using a NanoDrop 2000 spectrophotometer (Thermo Fisher Scientific, Massachusetts, USA). The crRNAs were stored at −80°C.

### Design and synthesis of p-crRNAs

We adapted the “R5-3PC” principle proposed by Hu’s group (20) to design p-RNAs, which completely bind the S region and partially bind with additional five bases of the R region of the crRNAs. A total of three PC linkers were then inserted in every six nucleotides in the p-RNAs and connected with two adjacent nucleotides through a short, UV-photocleavable C3 spacer arm. The structural diagram of the p-RNA can be found in Fig. S1. The synthesis of p-RNAs was conducted in Bio-LIFESCI Co., Ltd, and the sequences are available in Table S3.

For the preparation of p-crRNAs, a mixture of crRNA and p-RNA, crRNA (5 µL, 100 μM). and p-RNA (10 µL, 100 μM) was added into a 1× NEBuffer 2.1, and the volume was adjusted to 50 µL with RNase-free water. The mixture was heated to 70°C for 5 min, gradually cooling to room temperature at 0.6°C/min for complete annealing. The p-crRNA products were then stored at −20°C for later usage. The ratio of p-RNA and crRNA was set as 2:1 for better blocking efficiency.

### Development and evaluation of photocontrolled one-pot RPA/CRISPR-Cas12a assay

#### Photocontrolled CRISPR-Cas12a assay

Briefly, 0.2 µL of AsCas12a (10 µM), 0.4 µL of p-crRNA (10 µM), 1 µL of ssDNA reporter (10 µM), 1 µL of target DNA product amplified by PCR or RAA/RPA, and RNase-free water were mixed in 1 × NEBuffer 2.1 to reach to the final volume of 15 µL. The mixture was irradiated with a UV lamp (λ 365 nm, 35 W) for 30 s and then incubated at 37°C for 1 h. The fluorescence signals were recorded using a fluorescence detector (Qitian, Jiangsu, China). RPA was conducted by adding 29.5 µL of rehydration buffer into the RPA lyophilized pellet to make an RPA mixture. Then, 10 µL of amplification system containing 0.48 µL of each primer (10 µM), 5.9 µL of RPA mixture, 2 µL of DNA template, 0.5 µL of MgOAc (280 mM), and RNase-free water were added into a PCR tube and incubated at 39°C for 15 min.

#### Photocontrolled one-pot RPA/CRISPR-Cas12a assay

RPA system of 10 µL and CRISPR-Cas12a solution of 5 µL containing 1 µL of 10× NEBuffer 2.1, 0.2 µL of AsCas12a (10 µM), 0.4 µL of p-crRNA (10 µM), 1 µL of ssDNA reporter (10 µM), and RNase-free water were mixed and added into a PCR tube and incubated at 39°C for 15 min followed by irradiation for 30 s using 365 nm UV lighter. CRISPR-Cas12a detection was performed at 39°C for 40 min, while real-time fluorescence signals were recorded on a fluorescence detector (Qitian, Jiangsu, China).

### Statistical analysis

Data analysis was conducted using the IBM SPSS software, version 26 (IBM Corporation, Armonk, New York, USA). Two-tailed Student *t*-test was used to analyze the fluorescence difference between on- and off-target templates detected by photocontrolled one-pot RPA/CRISPR-Cas12a assay. Positive predictive value and negative predictive value as well as 95% binomial confidence intervals (CIs) were calculated according to the Clopper–Pearson score. The concordance between the photocontrolled one-pot assay and Sanger sequencing was calculated according to the kappa value. A *P*-value <0.05 was considered statistically significant. Data plotting was performed using the GraphPad Prism software (Version 8.0, La Jolla, California, USA)

## RESULTS

### Screening and evaluation of crRNAs

The CRISPR-Cas12a system has the capability to recognize single-nucleotide variation since the cleavage efficiency of Cas12a will be significantly impaired when there are mismatches between the crRNA and the target DNA sequence (28). Therefore, the CRISPR-Cas12a system has been widely used for molecular diagnosis and genotyping (29, 30). In this study, we tried to adapt the CRISPR-Cas12a system for rapid detection of major SARS-CoV-2 Omicron sub-lineages BA.1, BA.5.2, and BF.7. According to the sequence alignment results (Fig. S1), we first designed crRNAs-346T, crRNAs-486V, and crRNA-49X, which are specific for the three signature mutations in Omicron BA.1, BA.5.2, and BF.7. Among them, crRNA-49X has previously been demonstrated to be able to distinguish Omicron variants from alpha, beta, and delta variants and the wild-type strain of SARS-CoV-2 largely because it covers three mutation sites in the SARS-CoV-2 S gene (25, 26) (Fig. 2). Given that the number of mismatches between the crRNA and the target sequence, especially the mismatches in the seed region near PAM, may affect the specificity of the crRNA (31–33), we designed several new crRNAs (crRNA-346T-2,3,4) to increase the specificity of the original crRNA-346T-1 by introducing an extra mutation upstream of the mutation site (Fig. 2A). Furthermore, we designed crRNA-F486V-1, 2, and 4 containing suboptimal PAM sequences of GTTG, TTGC, and CCTT, respectively. Previous studies have shown that suboptimal PAM-mediated target sequence recognition can also activate Cas12a (34, 35). Due to the non-specific Cas12a activation caused by crRNA-486V-1, we designed crRNA-F486V-3 to contain an extra mutation in an attempt to enhance its specificity (Fig. 2B). Our results indicated that crRNA-346T-1 and crRNA-486V-2 showed the highest fluorescence ratio and are highly specific for the detection of Omicron BF.7 and BA.5.2 sub-lineage, respectively (Fig. 2C and D). Therefore, crRNA-346T-1, crRNA-486V-2, and crRNA-49X were used for further analysis.

### The key role of DTT in p-crRNA photoactivation

**Fig 3:**
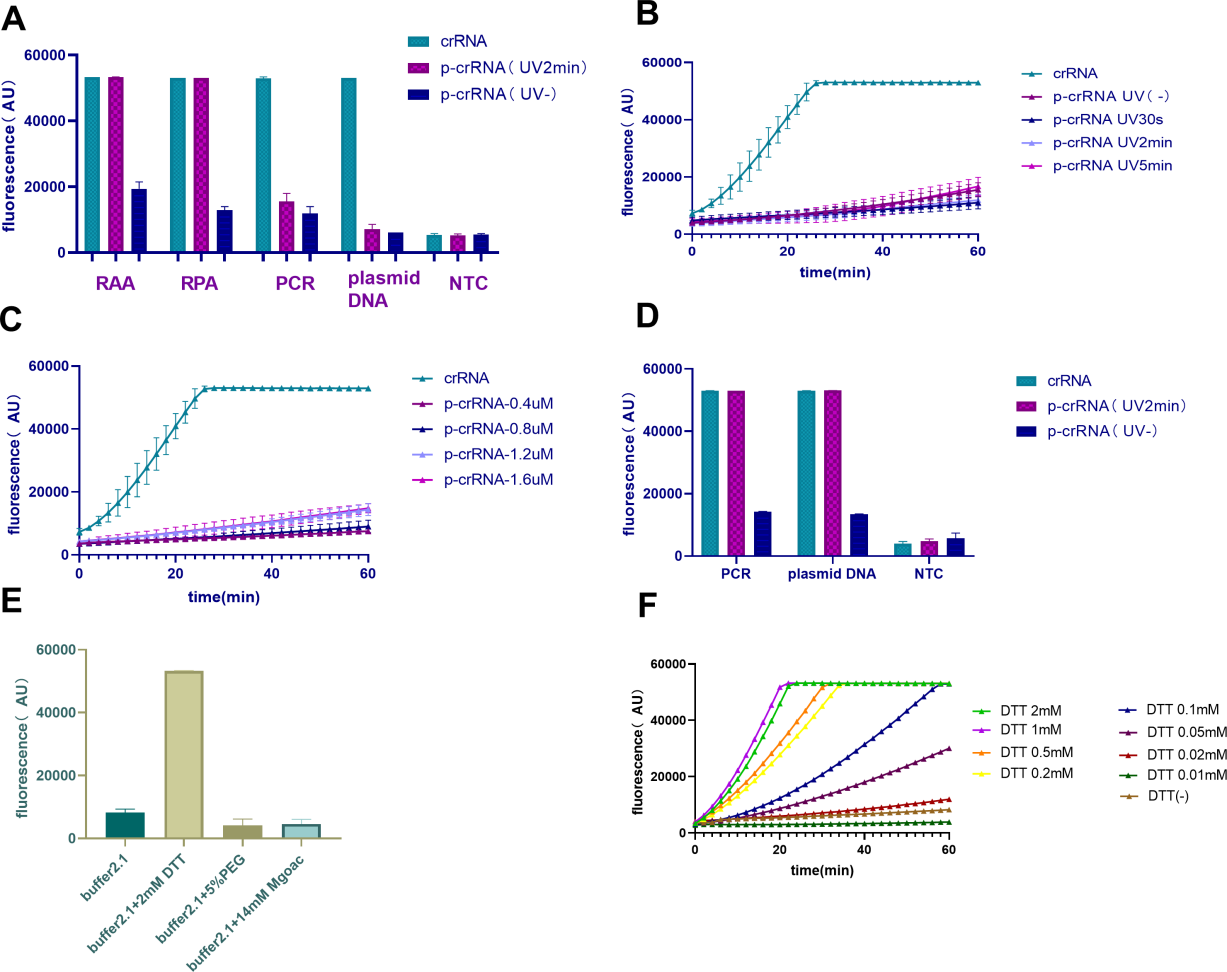
DTT is required for the cleavage of p-crRNA in photocontrolled CRISPR-Cas12a assay. (**A**) Omicron BA.1 plasmid DNA *per se* and its amplification products by RAA, RPA, and PCR could activate Cas12a to release fluorescence light (green bar) in the presence of crRNA using NEBuffer 2.1. However, only the amplification products of RAA and RPA, but not Omicron BA.1 plasmid DNA *per se* and its PCR products, could release fluorescence in the presence of photoactivated p-crRNA (purple bar). No fluorescence signal was obtained for the un-photoactivated p-crRNA (blue bar). Extending the photoactivation time (**B**) or increasing the amount of p-crRNA (**C**) could not activate Cas12a to release fluorescence signal in the presence of p-crRNA and PCR products of Omicron BA.1 plasmid DNA. However, PCR products were successfully cut in the presence of crRNA only (green line). (**D**) Omicron BA.1 plasmid DNA *per se* and its PCR products could activate Cas12a to release fluorescence in the presence of photoactivated p-crRNA (purple bar) when using RPA buffer to replace NEBuffer 2.1. (**E**) Cas12a could be activated to release fluorescence by adding DTT into NEBuffer 2.1 (yellow bar), but not PEG20000 (dark yellow) or MgOAc (green) or NEBuffer 2.1 *per se* (dark green). (**F**) The dose–response effect for different DTT concentrations was observed in photocontrolled CRISPR-Cas12a assay using PCR amplicons and UV irradiation for 2 min.

**Fig 4:**
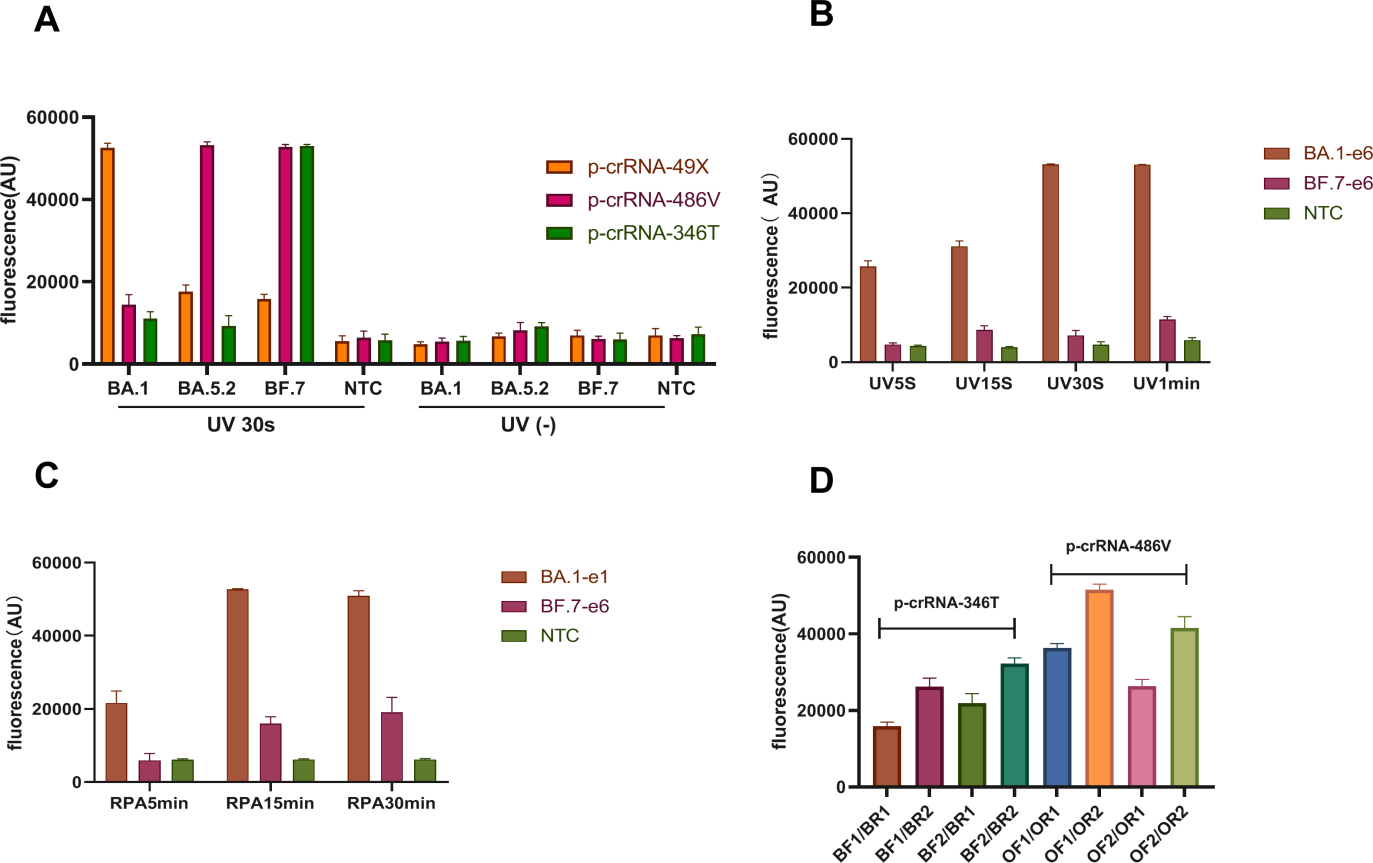
Photocontrolled one-pot RPA/CRISPR-Cas12a assay for the detection of SARS-CoV-2 Omicron mutants. (**A**) The role of UV excitation in the detection of Omicron plasmid (10^6^ copies/μL) of BA.1, BA.5.2, and BF.7. The detection performance varied by the photocleavage time (**B**) and the amplification time (**C**) when using p-crRNA49X for detecting BA.1 and BF.7, and RPA primer sets (**D**) when using p-crRNA-346T or p-crRNA-486V for detecting low-concentration plasmid template of BF.7 (-8e1 copies/μL). Fluorescence values were represented as mean ± standard deviation (SD) from three replicates. NTC, no template control.

We mixed RPA or RAA amplification products of Omicron BA.1 and photocontrolled CRISPR-Cas12a reagents containing p-crRNA-49X and irradiated the tube with a 365-nm UV lighter. As expected, fluorescence signal was recorded in the positive control, while no signal was observed for the negative control or un-irradiated tube (Fig. 3A), indicating that irradiation indeed cuts the p-crRNA-49X and dissociates p-RNA/crRNA complex, which in turn activates CRISPR-Cas12a. However, when PCR amplification products of Omicron BA.1 or Omicron BA.1 plasmid DNA in NEBuffer 2.1 were mixed with the aforementioned photocontrolled CRISPR-Cas12a reagents, no fluorescence signal was obtained (Fig. 3A), even extending UV irradiation time (Fig. 3B) or increasing the amounts of p-crRNA-49X (Fig. 3C). We changed PCR reagents but did not solve the problem (data not shown). Interestingly, replacing NEBuffer 2.1 with RPA buffer enabled the photoactivated CRISPR-Cas12a system to cleave both plasmid DNA and PCR amplicons (Fig. 4D), suggesting that the components in the RPA buffer may play a key role in the photoactivated CRISPR-Cas12a system.

After comparing the components of NEBuffer 2.1 and RPA buffer, we found that the major difference between the two buffers is DTT, MgOAc, and PEG20000 (Table S4). Then, we added DTT, MgOAc, and PEG20000 into NEBuffer 2.1 and explored the role of each component in the photoactivated CRISPR-Cas12a system. As seen in Fig. 3E, DTT played an important role, while MgOAc and PEG20000 showed a minor effect. Moreover, there was a dose–response relationship between DTT concentration and Cas12a activation, and 1–2 mM of DTT was optimal for the reaction (Fig. 3F).

### Development of photocontrolled one-pot RPA/CRISPR-Cas12a assay

A photocontrolled one-pot RPA/CRISPR-Cas12a assay was developed to include both RPA and CRISPR-Cas12a reagents for the detection of SARS-CoV-2 Omicron mutations using p-crRNA-49X and p-crRNA-346T that are specific for Omicron BA.1 and BF.7, respectively, and p-crRNA-486V targeting both BA.5.2 and BF.7 sub-lineages. The assay system integrated amplification, photolysis, Cas12a activation, and detection and could successfully distinguish the three Omicron sub-lineages based on the strength of fluorescence signals in one tube reaction (Fig. 4A). We further optimized the reaction conditions and found that 15 min of RPA amplification was sufficient for NAA, while a 30-s UV light irradiation was enough for releasing crRNA from p-crRNA (Fig. 4B) and for producing amplicons for further CRISPR detection, even for as low as 20 copies/μL of DNA template (Fig. 4C). Additionally, the design of RPA primers was also vital for minimizing nonspecific amplification and enhancing detection sensitivity, especially for low copy templates. The primers were designed to target the conserved sequences of the SARS-CoV-2 spike gene, and the secondary structure and primer dimer formation were assessed using the OligoAnalyzer Tool of Integrated DNA Technologies. We found that the primer sets of BF2/BR2 and OF1/OR2 worked best for the amplification of the fragments specific for R346T and F486V/49X mutations, respectively (Fig. 4D).

### Evaluation of one-pot RPA/CRISPR-Cas12a assay

**Fig 5:**
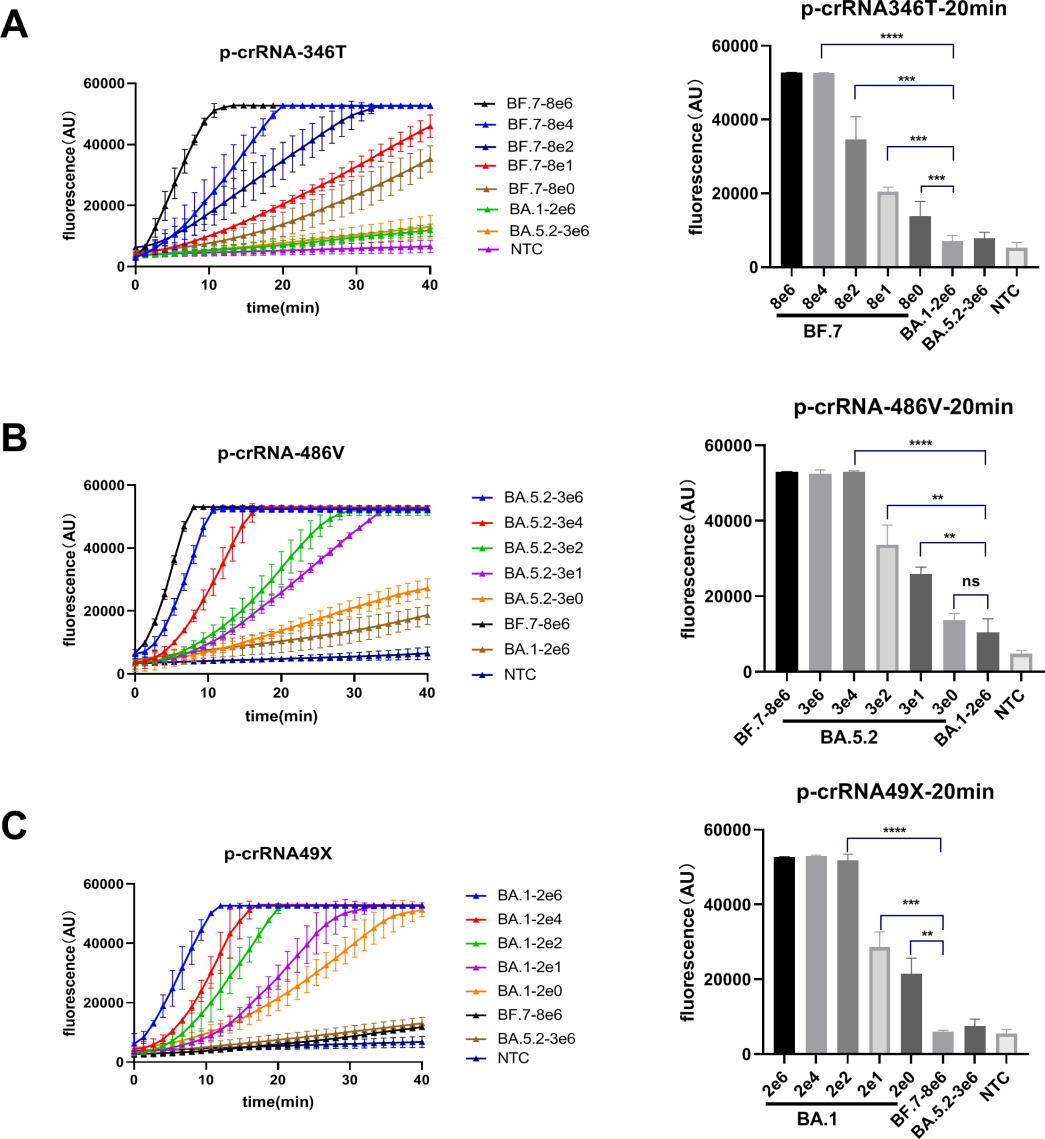
LOD of photocontrolled one-pot RPA/CRISPR-Cas12a for detecting Omicron plasmids of BF.7, BA.5.2, and BA.1 using p-crRNA-346T (**A**), p-crRNA-486V (**B**), and p-crRNA-49X (**C**), respectively. The real-time reaction curves are presented on the left panels and the quantitation results on the right panels. Data from three repeats are represented as mean ± standard deviation (SD). NTC refers to no template control. Two-tailed Student’s *t*-test was used to analyze the fluorescence difference between on- and off-target template. ns, *P* > 0.05; **P < 0.01; ***P < 0.001; ****P < 0.0001. NTC, no template control.

Based on the optimized conditions, we determined the low limit of detection (LOD) of the photocontrolled one-pot RPA/CRISPR-Cas12a assay through serial dilutions of the Omicron plasmid DNAs, which was 8 copies/μL for BF.7 (Fig. 5A), 30 copies/μL for BA.5.2 (Fig. 5B), and 2 copies/μL for BA.1 (Fig. 5C), respectively, within 20 min of CRISPR-Cas12a detection. These LOD values are comparable to those achieved by the conventional two-step RPA/CRISPR-Cas12a assay (20, 36), highlighting the successful development of photocontrolled one-pot RPA/CRISPR-Cas12a assay.

**Fig 6:**
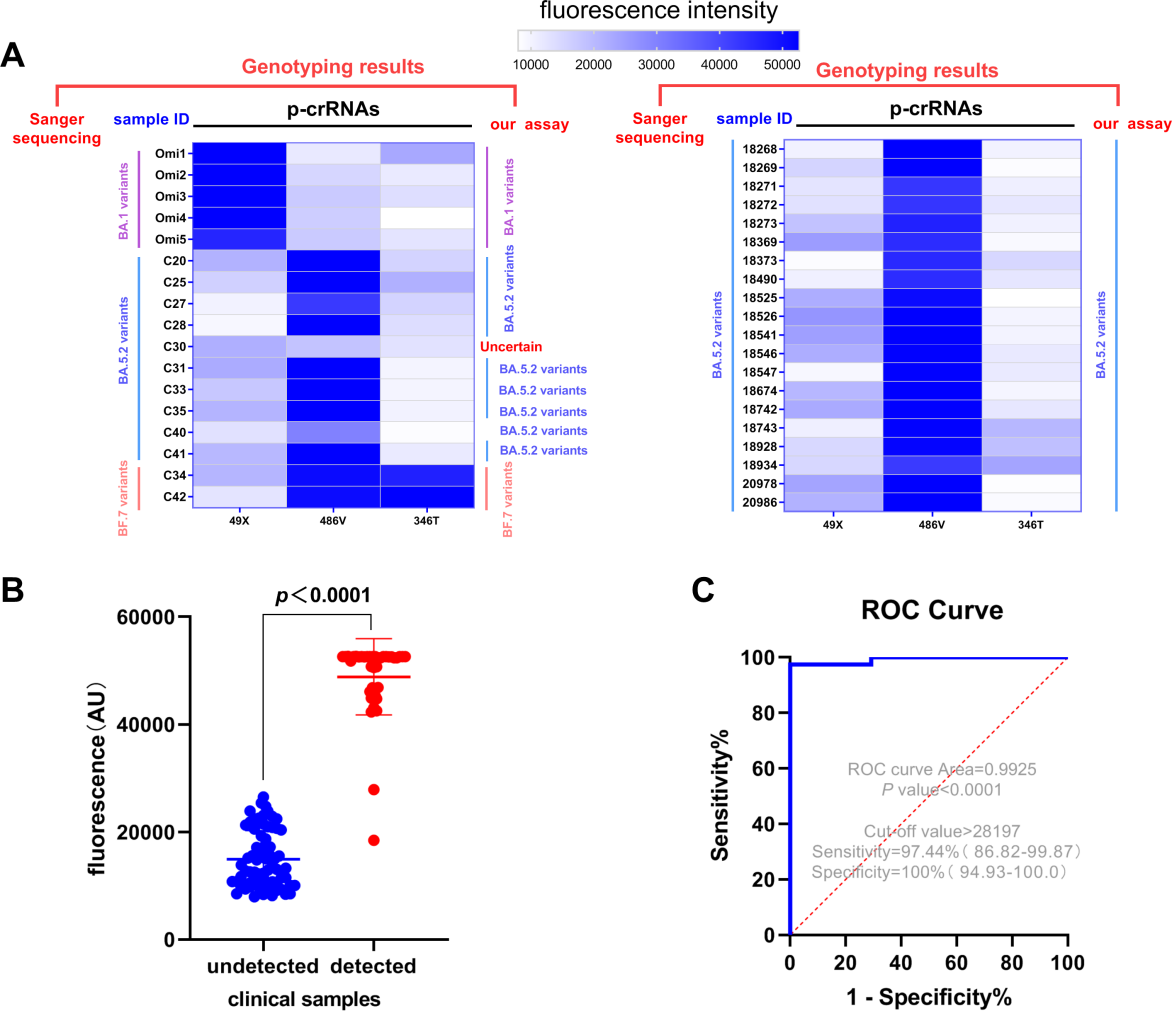
Performance of the photocontrolled one-pot RPA/CRISPR-Cas12a assay for detection of SARS-CoV-2 clinical samples. (**A**) Heat map shows the testing results of 37 clinical samples infected with SARS-CoV-2 Omicron variant by RPA-CRISPR/Cas12a assay and comparison with the genotyping results of Sanger sequencing. The strength of fluorescence signal is depicted using a color scale bar (**A**) and presented as mean ± SD for the clinical samples that should be detected (red) and undetected (blue), respectively. (**B**) The performance of one-pot RPA/CRISPR-Cas12a assay was evaluated by an ROC curve (**C**).

**Fig 7:**
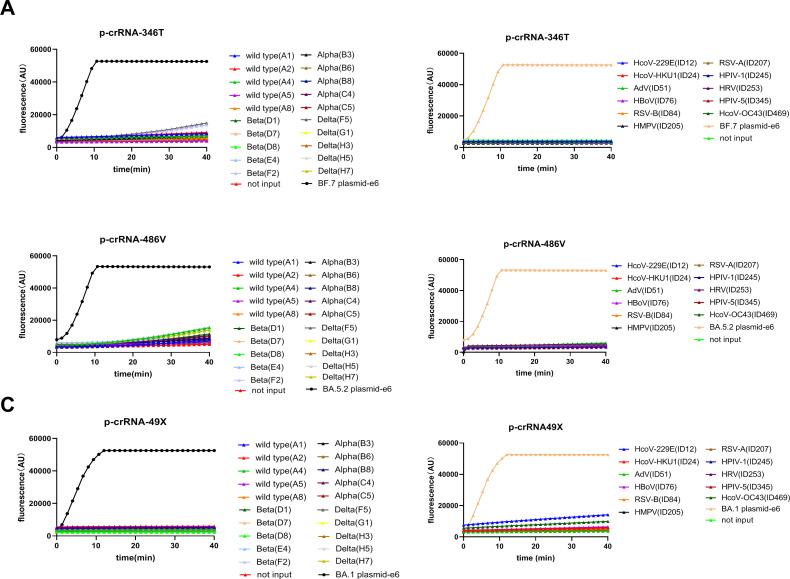
The specificity of the photocontrolled one-pot RPA/CRISPR-Cas12a assay. It was evaluated by detecting previously prevalent SARS-CoV-2 strains, including 5 wild-type strains, 5 alpha variants, 5 beta variants, and 5 delta variants (left panel) and 11 other coronaviruses or human respiratory viruses, including HcoV-229E, HcoV-HKU1, Adv, HBoV, RSV-B, HMPV, RSV-A, HPIV-1, HRV, HPIV-5, and HcoV-OC43 (right panel), using p-crRNA-346T (**A**), p-crRNA-486V (**B**), and p-crRNA-49X (**C**), respectively.

The capability of the three p-crRNAs to distinguish different Omicron sub-lineages was further analyzed in 37 clinical samples infected with SARS-CoV-2 Omicron variants, including 5 samples infected with BA.1 variants, 30 with BA.5.2 variants, and 2 with BF.7 variants, and the results were compared with Sanger sequencing results (Fig. 6A). Both p-crRNA-49X and 346T showed expected results. Only two samples (C30 and C40) of BA.5.2 exhibited weak fluorescence signals (18,463 and 29,875, respectively) when using p-crRNA-486V. In contrast, the fluorescence units for the samples that are expected to be detected and undetected were 50,242 ± 3,628 and 14,954 ± 52,88, respectively (*P* < 0.0001, Fig. 6B). The receiver operating characteristic (ROC) curve showed that at the fluorescence threshold of 28,197, the ROC area was 0.9925, whereas the sensitivity and specificity were 97.44% (95% CI: 86.82–99.87%) and 100% (95% CI: 94.93–100%), respectively (Fig. 6C). For 20 SARS-CoV-2 Omicron-negative clinical samples, including 5 wild-type strains, 5 alpha variants, 5 beta variants, and 5 delta variants of SARS-CoV-2, and 11 samples infected with other respiratory pathogens, no cross-reactivity was observed for any of the p-crRNAs (Fig. 7A through C). Compared with Sanger sequencing, one-pot RPA/CRISPR-Cas12a assay showed a specificity of 100% and a high sensitivity of 97.30% (Table 1), while the two assays showed a concordance of 98.25% (56/57) and a kappa value of 0.962 (Table 1).

**TABLE 1:** Concordance between one-pot RPA/CRISPR-Cas12a assay and Sanger sequencing^*a*^

One-pot RPA-CRISPR assay	Sequencing results		Sensitivity (%, 95% CI)	Specificity (%, 95% CI)	Positive predictive value (%, 95% CI)	Negative predictive value (%, 95% CI)	Kappa value
	Positive	Negative					
Positive	36	0	97.30 (85.84–99.93)	100.00 (83.16–100.00)	100.00 (90.26–100.00)	99.11 (94.14–99.87)	0.962
Negative	1	20					

^*a*^ The prevalence of COVID-19 is assumed to be 25% when calculating the positive and negative predictive values.

## DISCUSSION

It is ideal and critical to complete NAA and CRISPR-Cas12a detection in one tube to make the detection simple and rapid and to decrease the risk of contamination. We have previously reported a one-tube reaction system by putting RPA reagents at the bottom of the PCR tube and CRISPR-Cas12a reagents at the lid of the PCR tube to physically separate the two reaction components (26). After template amplification, CRISPR-Cas12a reagents were spun down to mix with the amplified products and initiate CRISPR-Cas12a-mediated detection. In the current study, we tried to use photoactivated p-crRNA to precisely control the time of CRISPR-Cas12a activation, but not interfering NAA in the presence of both RPA and CRISPR-Cas12a reagents. It has been reported that this photoactivatable crRNA strategy was successfully applied to SARS-CoV-2 RNA detection (20).

Our study demonstrated the feasibility of the photoactivatable crRNA strategy in developing one-pot nucleic acid amplification and CRISPR-Cas detection system. Although there are different ways to develop one-pot NAA/CRISPR-Cas assay, the photocontrolled method provides us another option and can be easily incorporated into the isothermal amplification system, especially in a portable detection device with small modification. In addition, the current photocontrolled technique makes it possible to precisely manipulate both NAA and CRISPR-Cas detection, which is an advantage for optimizing the assay conditions separately.

We must emphasize that the development of the photocontrolled one-pot NAA/CRISPR-Cas assay is not simple and straightforward. Our results showed that even DTT and its concentration can significantly affect the assay efficiency and performance. DTT is commonly used as a reducing agent to disrupt disulfide bonds in proteins and to maintain their proper conformation (37, 38). In RPA and RAA kits, DTT is used to optimize the activity of recombinase enzymes and to enhance the stability of DNA templates (39, 40). In addition, DTT has been used to boost the activity of Cas12a in the CRISPR system (41). Our results indicated that the addition of DTT may help maintain appropriate structure or conformation of crRNA/Cas12a for efficient photolysis of p-crRNA.

Other critical factors include the design of both p-RNA and crRNA and the conditions for the two reactions. We found that few nucleotide mismatches between the crRNA and the target gene could still activate Cas to cleave the template, resulting in false-positive detection. In addition, our results indicate that the assay specificity could be affected by suboptimal PAM sequences in crRNAs, while the introduction of additional mutations in crRNAs may be needed to improve the assay sensitivity and specificity. Unfortunately, it is difficult to predict the location and number of the introduced mutations, which require a careful design of crRNA and a large amount of screening. Therefore, assay optimization includes screening of the best crRNAs, refining assay conditions, including reaction time, and setting a fluorescence threshold according to the ROC curve. In our study, the fluorescence signal is usually collected within 10–20 min during CRISPR-Cas detection when the background activity is still low. However, it is still a challenge for the accurate diagnosis of weakly positive clinical samples.

While our photocontrolled one-pot assay showed promising specificity and sensitivity with a substantial concordance with Sanger sequencing, further validation with larger clinical samples is necessary. To further simplify the detection system, the assay should integrate with nucleic acid extraction or purification and reverse transcription, which can be achieved in an automatic detection system or using a microfluidic device (42, 43). Moreover, the current method primarily targeted a limited number of mutation sites; future efforts can focus on developing high-throughput multiplex screening assays (44).

### Conclusion

We proved the feasibility of photoactivated strategy for developing one-pot RPA/CRISPR-Cas12a assay. Compared with Sanger sequencing, RT-qPCR, and the traditional two-step NAA/CRISPR-Cas detection assay, one-pot RPA/CRISPR-Cas12a assay is a simple, rapid method without contamination risk. It can be developed as a point-of-care testing and implemented in resource-limiting settings without the need of complex equipment and facilities.

## SUPPLEMENTAL MATERIAL

Tables S1 to S4 and Fig. S1 and S2 spectrum.03645-23-s0001.pdf Nucleotide sequence and sequence alignment.

ASM does not own the copyrights to Supplemental Material that may be linked to, or accessed through, an article. The authors have granted ASM a non-exclusive, world-wide license to publish the Supplemental Material files. Please contact the corresponding author directly for reuse.

## References

[1] Pickar-Oliver A, Gersbach CA. 2019. The next generation of CRISPR-Cas technologies and applications. Nat Rev Mol Cell Biol 20:490–507. doi:10.1038/s41580-019-0131-5 31147612 PMC7079207

[2] Chertow DS. 2018. Next-generation diagnostics with CRISPR. Science 360:381–382. doi:10.1126/science.aat4982 29700254

[3] Wang X, Zhong M, Liu Y, Ma P, Dang L, Meng Q, Wan W, Ma X, Liu J, Yang G, Yang Z, Huang X, Liu M. 2020. Rapid and sensitive detection of COVID-19 using CRISPR/Cas12a-based detection with naked eye readout, CRISPR/Cas12a-NER. Science Bulletin 65:1436–1439. doi:10.1016/j.scib.2020.04.041 32373393 PMC7198415

[4] de Puig H, Lee RA, Najjar D, Tan X, Soeknsen LR, Angenent-Mari NM, Donghia NM, Weckman NE, Ory A, Ng CF, Nguyen PQ, Mao AS, Ferrante TC, Lansberry G, Sallum H, Niemi J, Collins JJ. 2021. Minimally instrumented SHERLOCK (miSHERLOCK) for CRISPR-based point-of-care diagnosis of SARS-CoV-2 and emerging variants. Sci Adv 7:32. doi:10.1126/sciadv.abh2944 PMC8346217 34362739

[5] Zhou Y, Zhang L, Xie YH, Wu J. 2022. Advancements in detection of SARS-CoV-2 infection for confronting COVID-19 pandemics. Lab Invest 102:4–13. doi:10.1038/s41374-021-00663-w 34497366 PMC8424153

[6] Afzal A. 2020. Molecular diagnostic technologies for COVID-19: limitations and challenges. J Adv Res 26:149–159. doi:10.1016/j.jare.2020.08.002 32837738 PMC7406419

[7] Chiara M, D’Erchia AM, Gissi C, Manzari C, Parisi A, Resta N, Zambelli F, Picardi E, Pavesi G, Horner DS, Pesole G. 2021. Next generation sequencing of SARS-CoV-2 genomes: challenges, applications and opportunities. Brief Bioinform 22:616–630. doi:10.1093/bib/bbaa297 33279989 PMC7799330

[8] Kellner MJ, Koob JG, Gootenberg JS, Abudayyeh OO, Zhang F. 2019. SHERLOCK: nucleic acid detection with CRISPR nucleases. Nat Protoc 14:2986–3012. doi:10.1038/s41596-019-0210-2 31548639 PMC6956564

[9] Chen JS, Ma E, Harrington LB, Da Costa M, Tian X, Palefsky JM, Doudna JA. 2018. CRISPR-Cas12a target binding unleashes indiscriminate single-stranded DNase activity. Science 360:436–439. doi:10.1126/science.aar6245 29449511 PMC6628903

[10] Ali Z, Aman R, Mahas A, Rao GS, Tehseen M, Marsic T, Salunke R, Subudhi AK, Hala SM, Hamdan SM, Pain A, Alofi FS, Alsomali A, Hashem AM, Khogeer A, Almontashiri NAM, Abedalthagafi M, Hassan N, Mahfouz MM. 2020. iSCAN: an RT-LAMP-coupled CRISPR-Cas12 module for rapid, sensitive detection of SARS-CoV-2. Virus Res. 288:198129. doi:10.1016/j.virusres.2020.198129 32822689 PMC7434412

[11] Wang B, Wang R, Wang D, Wu J, Li J, Wang J, Liu H, Wang Y. 2019. Cas12aVDet: a CRISPR/Cas12a-based platform for rapid and visual nucleic acid detection. Anal Chem 91:12156–12161. doi:10.1021/acs.analchem.9b01526 31460749

[12] Pang B, Xu J, Liu Y, Peng H, Feng W, Cao Y, Wu J, Xiao H, Pabbaraju K, Tipples G, Joyce MA, Saffran HA, Tyrrell DL, Zhang H, Le XC. 2020. Isothermal amplification and ambient visualization in a single tube for the detection of SARS-CoV-2 using loop-mediated amplification and CRISPR technology. Anal Chem 92:16204–16212. doi:10.1021/acs.analchem.0c04047 33238709

[13] Lin M, Yue H, Tian T, Xiong E, Zhu D, Jiang Y, Zhou X. 2022. Glycerol additive boosts 100-fold sensitivity enhancement for one-pot RPA-CRISPR/Cas12a assay. Anal Chem. 94:8277–8284. doi:10.1021/acs.analchem.2c00616 35635176

[14] Yin K, Ding X, Li Z, Zhao H, Cooper K, Liu C. 2020. Dynamic aqueous multiphase reaction system for one-pot CRISPR-Cas12a-based ultrasensitive and quantitative molecular diagnosis. Anal Chem 92:8561–8568. doi:10.1021/acs.analchem.0c01459 32390420 PMC7588651

[15] Lu S, Tong X, Han Y, Zhang K, Zhang Y, Chen Q, Duan J, Lei X, Huang M, Qiu Y, Zhang DY, Zhou X, Zhang Y, Yin H. 2022. Fast and sensitive detection of SARS-CoV-2 RNA using suboptimal protospacer adjacent motifs for Cas12a. Nat Biomed Eng 6:286–297. doi:10.1038/s41551-022-00861-x 35314803

[16] Ding X, Yin K, Li Z, Lalla RV, Ballesteros E, Sfeir MM, Liu C. 2020. Ultrasensitive and visual detection of SARS-CoV-2 using all-in-one dual CRISPR-Cas12a assay. Nat Commun 11:4711. doi:10.1038/s41467-020-18575-6 32948757 PMC7501862

[17] Jain PK, Ramanan V, Schepers AG, Dalvie NS, Panda A, Fleming HE, Bhatia SN. 2016. Development of light-activated CRISPR using guide RNAs with photocleavable protectors. Angew Chem Int Ed Engl 55:12440–12444. doi:10.1002/anie.201606123 27554600 PMC5864249

[18] Zou RS, Liu Y, Wu B, Ha T. 2021. Cas9 deactivation with photocleavable guide RNAs. Mol Cell 81:1553–1565. doi:10.1016/j.molcel.2021.02.007 33662274 PMC8026597

[19] Chen Y, Xu X, Wang J, Zhang Y, Zeng W, Liu Y, Zhang X. 2022. Photoactivatable CRISPR/Cas12a strategy for one-pot DETECTR molecular diagnosis. Anal Chem. 94:9724–9731. doi:10.1021/acs.analchem.2c01193 35762828

[20] Hu M, Qiu Z, Bi Z, Tian T, Jiang Y, Zhou X. 2022. Photocontrolled crRNA activation enables robust CRISPR-Cas12a diagnostics. Proc Natl Acad Sci U S A 119:e2202034119. doi:10.1073/pnas.2202034119 35727982 PMC9245704

[21] Hu M, Liu R, Qiu Z, Cao F, Tian T, Lu Y, Jiang Y, Zhou X. 2023. Light-start CRISPR-Cas12a reaction with caged crRNA enables rapid and sensitive nucleic acid detection. Angew Chem Int Ed Engl 62:e202300663. doi:10.1002/anie.202300663 37016515

[22] Bal A, Destras G, Gaymard A, Stefic K, Marlet J, Eymieux S, Regue H, Semanas Q, d’Aubarede C, Billaud G, Laurent F, Gonzalez C, Mekki Y, Valette M, Bouscambert M, Gaudy-Graffin C, Lina B, Morfin F, Josset L, COVID-Diagnosis HCL Study Group. 2021. Two-step strategy for the identification of SARS-CoV-2 variant of concern 202012/01 and other variants with spike deletion H69-V70. Euro Surveill 26:3. doi:10.2807/1560-7917.ES.2021.26.3.2100008 PMC7848679 33478625

[23] He C, Lin C, Mo G, Xi B, Li AA, Huang D, Wan Y, Chen F, Liang Y, Zuo Q, Xu W, Feng D, Zhang G, Han L, Ke C, Du H, Huang L. 2022. Rapid and accurate detection of SARS-CoV-2 mutations using a Cas12a-based sensing platform. Biosens Bioelectron 198:113857. doi:10.1016/j.bios.2021.113857 34894625 PMC8635686

[24] Fasching CL, Servellita V, McKay B, Nagesh V, Broughton JP, Sotomayor-Gonzalez A, Wang B, Brazer N, Reyes K, Streithorst J, Deraney RN, Stanfield E, Hendriks CG, Fung B, Miller S, Ching J, Chen JS, Chiu CY. 2022. COVID-19 variant detection with a high-fidelity CRISPR-Cas12 enzyme. J Clin Microbiol 60:e0026122. doi:10.1128/jcm.00261-22 35766492 PMC9297821

[25] Liang Y, Lin H, Zou L, Deng X, Tang S. 2022. Rapid detection and tracking of Omicron variant of SARS-CoV-2 using CRISPR-Cas12a-based assay. Biosens Bioelectron 205:114098. doi:10.1016/j.bios.2022.114098 35189535 PMC8849905

[26] Lin H, Liang Y, Zou L, Li B, Zhao J, Wang H, Sun J, Deng X, Tang S. 2022. Combination of isothermal recombinase-aided amplification and CRISPR-Cas12a-mediated assay for rapid detection of major severe acute respiratory syndrome coronavirus 2 variants of concern. Front Microbiol 13:945133. doi:10.3389/fmicb.2022.945133 35836420 PMC9274097

[27] Liang Y, Lin H, Zou L, Zhao J, Li B, Wang H, Lu J, Sun J, Yang X, Deng X, Tang S. 2021. CRISPR-Cas12a-based detection for the major SARS-CoV-2 variants of concern. Microbiol Spectr 9:e0101721. doi:10.1128/Spectrum.01017-21 34787487 PMC8597640

[28] Li SY, Cheng QX, Wang JM, Li XY, Zhang ZL, Gao S, Cao RB, Zhao GP, Wang J. 2018. CRISPR-Cas12a-assisted nucleic acid detection. Cell Discov 4:20. doi:10.1038/s41421-018-0028-z 29707234 PMC5913299

[29] Broughton JP, Deng X, Yu G, Fasching CL, Servellita V, Singh J, Miao X, Streithorst JA, Granados A, Sotomayor-Gonzalez A, Zorn K, Gopez A, Hsu E, Gu W, Miller S, Pan CY, Guevara H, Wadford DA, Chen JS, Chiu CY. 2020. CRISPR-Cas12-based detection of SARS-CoV-2. Nat Biotechnol 38:870–874. doi:10.1038/s41587-020-0513-4 32300245 PMC9107629

[30] Liang Y, Zou L, Lin H, Li B, Zhao J, Wang H, Sun J, Chen J, Mo Y, Yang X, Deng X, Tang S. 2022. Detection of major SARS-CoV-2 variants of concern in clinical samples via CRISPR-Cas12a-mediated mutation-specific assay. ACS Synth Biol 11:1811–1823. doi:10.1021/acssynbio.1c00643 35481381

[31] Yamano T, Nishimasu H, Zetsche B, Hirano H, Slaymaker IM, Li Y, Fedorova I, Nakane T, Makarova KS, Koonin EV, Ishitani R, Zhang F, Nureki O. 2016. Crystal structure of Cpf1 in complex with guide RNA and target DNA. Cell 165:949–962. doi:10.1016/j.cell.2016.04.003 27114038 PMC4899970

[32] Huang X, Zhang F, Zhu K, Lin W, Ma W. 2021. dsmCRISPR: dual synthetic mismatches CRISPR/Cas12a-based detection of SARS-CoV-2 D614G mutation. Virus Res. 304:198530. doi:10.1016/j.virusres.2021.198530 34363850 PMC8339451

[33] Meng Q, Wang X, Wang Y, Dang L, Liu X, Ma X, Chi T, Wang X, Zhao Q, Yang G, Liu M, Huang X, Ma P. 2021. Detection of the SARS-CoV-2 D614G mutation using engineered Cas12a guide RNA. Biotechnol J 16:e2100040. doi:10.1002/biot.202100040 33595922

[34] Yamano T, Zetsche B, Ishitani R, Zhang F, Nishimasu H, Nureki O. 2017. Structural basis for the canonical and non-canonical PAM recognition by CRISPR-Cpf1. Mol Cell 67:633–645. doi:10.1016/j.molcel.2017.06.035 28781234 PMC5957536

[35] Misiurina MA, Chirinskaite AV, Fotina AS, Zelinsky AA, Sopova JV, Leonova EI. 2022. New PAM improves the single-base specificity of crRNA-guided LbCas12a nuclease. Life (Basel) 12:11. doi:10.3390/life12111927 PMC9698171 36431062

[36] Mayuramart O, Nimsamer P, Rattanaburi S, Chantaravisoot N, Khongnomnan K, Chansaenroj J, Puenpa J, Suntronwong N, Vichaiwattana P, Poovorawan Y, Payungporn S. 2021. Detection of severe acute respiratory syndrome coronavirus 2 and influenza viruses based on CRISPR-Cas12a. Exp Biol Med (Maywood) 246:400–405. doi:10.1177/1535370220963793 33153299 PMC7885046

[37] Getz EB, Xiao M, Chakrabarty T, Cooke R, Selvin PR. 1999. A comparison between the sulfhydryl reductants tris(2-carboxyethyl)phosphine and dithiothreitol for use in protein biochemistry. Anal Biochem 273:73–80. doi:10.1006/abio.1999.4203 10452801

[38] Netto LE, Stadtman ER. 1996. The iron-catalyzed oxidation of dithiothreitol is a biphasic process: hydrogen peroxide is involved in the initiation of a free radical chain of reactions. Arch Biochem Biophys 333:233–242. doi:10.1006/abbi.1996.0386 8806776

[39] Park JS, Wang M, Park SJ, Lee SH. 1999. Zinc finger of replication protein A, a non-DNA binding element, regulates its DNA binding activity through redox. J Biol Chem 274:29075–29080. doi:10.1074/jbc.274.41.29075 10506160

[40] Munawar MA. 2022. Critical insight into recombinase polymerase amplification technology. Expert Rev Mol Diagn 22:725–737. doi:10.1080/14737159.2022.2109964 35950726

[41] Yue H, Shu B, Tian T, Xiong E, Huang M, Zhu D, Sun J, Liu Q, Wang S, Li Y, Zhou X. 2021. Droplet Cas12a assay enables DNA quantification from unamplified samples at the single-molecule level. Nano Lett. 21:4643–4653. doi:10.1021/acs.nanolett.1c00715 34038136

[42] Li Z, Ding X, Yin K, Avery L, Ballesteros E, Liu C. 2022. Instrument-free, CRISPR-based diagnostics of SARS-CoV-2 using self-contained microfluidic system. Biosens Bioelectron 199:113865. doi:10.1016/j.bios.2021.113865 34906838 PMC8653405

[43] Sun Z, Lin KF, Zhao ZH, Wang Y, Hong XX, Guo JG, Ruan QY, Lu LY, Li X, Zhang R, Yang CY, Li BA. 2022. An automated nucleic acid detection platform using digital microfluidics with an optimized Cas12a system. Sci China Chem 65:630–640. doi:10.1007/s11426-021-1169-1 35126481 PMC8809245

[44] Welch NL, Zhu M, Hua C, Weller J, Mirhashemi ME, Nguyen TG, Mantena S, Bauer MR, Shaw BM, Ackerman CM, et al.. 2022. Multiplexed CRISPR-based microfluidic platform for clinical testing of respiratory viruses and identification of SARS-CoV-2 variants. Nat Med 28:1083–1094. doi:10.1038/s41591-022-01734-1 35130561 PMC9117129

