# Supplementary material for: A photocontrolled one-pot isothermal amplification and CRISPR-Cas12a assay for rapid detection of SARS-CoV-2 Omicron variants
Source: Microbiol Spectr. 2024 Feb 6;12(3):e03645-23. doi: 10.1128/spectrum.03645-23 (PMC10913417; doi:10.1128/spectrum.03645-23)
Supplement: Tables S1 to S4 and Fig. S1 and S2 — Nucleotide sequence and sequence alignment. [file spectrum.03645-23-s0001.pdf]

Table S1. Summary of DNA target sequences used for plasmid construction

Omicron<br>BA.1<br>S gene	ATGTTTGTTTTTCTTGTTTTATTGCCACTAGTCTCTAGTCAGTGTGTTAATCTTAC<br>AACCAGAACTCAATTACCCCCTGCATACACTAATTCTTTCACACGTGGTGTTTAT<br>TACCCTGACAAAGTTTTCAGATCCTCAGTTTTACATTCAACTCAGGACTTGTTCT<br>TACCTTTCTTTTCCAATGTTACTTGGTTCCATGTTATACATGTCTCTGGGACCAAT<br>GGTACTAAGAGGTTTGATAACCCTGTCCTACCATTTAATGATGGTGTTTATTTTGC<br>TTCCATTGAGAAGTCTAACATAATAAGAGGCTGGATTTTTGGTACTACTTTAGATT<br>CGAAGACCCAGTCCCTACTTATTGTTAATAACGCTACTAATGTTGTTATTAAAGTC<br>TGTGAATTTCAATTTTGTAATGATCCATTTTTGGGTGTTTATGACCACAAAAACA<br>ACAAAAGTTGGATGGAAAGTGAGTTCAGAGTTTATTCTAGTGCGAATAATTGCA<br>CTTTTGAATATGTCTCTCAGCCTTTTCTTATGGACCTTGAAGGAAAACAGGGTAA<br>TTTCAAAAATCTTAGGGAATTTGTGTTTAAGAATATTGATGGTTATTTTAAAATAT<br>ATTCTAAGCACACGCCTATTAATATAGTGCGTGATCTCCCTCAGGGTTTTTCGGCT<br>TTAGAACCATTGGTAGATTTGCCAATAGGTATTAACATCACTAGGTTTCAAACT<br>TTACTTGCTTTACATAGAAGTTATTTGACTCCTGGTGATTCTTCTTCAGGTTGGAC<br>AGCTGGTGCTGCAGCTTATTATGTGGGTTATCTTCAACCTAGGACTTTTCTATTAA<br>AATATAATGAAAATGGAACCATTACAGATGCTGTAGACTGTGCACTTGACCCTCT<br>CTCAGAAACAAAGTGTACGTTGAAATCCTTCACTGTAGAAAAAGGAATCTATCA<br>AACTTCTAACTTTAGAGTCCAACCAACAGAATCTATTGTTAGATTTCCTAATATTA<br>CAAACTTGTGCCCTTTTGATGAAGTTTTTAACGCCACCAGATTTGCATCTGTTTA<br>TGCTTGGAACAGGAAGAGAATCAGCAACTGTGTTGCTGATTATTCTGTCCTATAT<br>AATCTCGCACCATTTTTCACTTTTAAGTGTTATGGAGTGTCTCCTACTAAATTAAA<br>TGATCTCTGCTTTACTAATGTCTATGCAGATTCATTTGTAATTAGAGGTGATGAAG<br>TCAGACAAATCGCTCCAGGGCAAACTGGAAATATTGCTGATTATAATTATAAATT<br>ACCAGATGATTTTACAGGCTGCGTTATAGCTTGGAATTCTAACAAACTTGATTCT<br>AAGGTTAGTGGTAATTATAATTACCTGTATAGATTGTTTAGGAAGTCTAATCTCAA<br>ACCTTTTGAGAGAGATATTTCAACTGAAATCTATCAGGCCGGTAACAAACCTTGT<br>AATGGTGTTGCAGGTTTTAATTGTTACTTTCCTTTACGATCATATAGTTTCCGACC<br>CACTTATGGTGTTGGTCACCAACCATACAGAGTAGTAGTACTTTCTTTTGAACTT<br>CTACATGCACCAGCAACTGTTTGTGGACCTAAAAAGTCTACTAATTTGGTTAAA<br>AACAAATGTGTCAATTTCAACTTCAATGGTTTAAAAGGCACAGGTGTTCTTACT<br>GAGTCTAACAAAAAGTTTCTGCCTTTCCAACAATTTGGCAGAGACATTGCTGAC<br>ACTACTGATGCTGTCCGTGATCCACAGACACTTGAGATTCTTGACAT<br>TACACCATGTTCTTTTGGTGGTGTCAGTGTTATAACACCAGGAACAAATACTTCT<br>AACCAGGTTGCTGTTCTTTATCAGGGTGTTAACTGCACAGAAGTCCCTGTTGCTA<br>TTCATGCAGATCAACTTACTCCTACTTGGCGTGTTTATTCTACAGGTTCTAATGTT<br>TTTCAAACACGTGCAGGCTGTTTAATAGGGGCTGAATATGTCAACAACTCATATG<br>AGTGTGACATACCCATTGGTGCAGGTATATGCGCTAGTTATCAGACTCAGACTAA<br>ATCTCATCGGCGGGCACGTAGTGTAGCTAGTCAATCCATCATTGCCTACACTATG<br>TCACTTGGTGCAGAAAATTCAGTTGCTTACTCTAATAACTCTATTGCCATACCCA<br>CAAATTTTACTATTAGTGTTACCACAGAAATTCTACCAGTGTCTATGACCAAG
Omicron<br>BA.5.2<br>S gene	TCTTCAGGTTGGACAGCTGGTGCTGCAGCTTATTATGTGGGTTATCTTCAAC<br>CTAGGACTTTTCTATTAAAATATAATGAAAATGGAACCATTACAGATGCTGTA<br>GACTGTGCACTTGACCCTCTCTCAGAAACAAAGTGTACGTTGAAATCCTTC

	ACTGTAGAAAAAGGAATCTATCAAACTTCTAACTTTAGAGTCCAACCAACA<br>GAATCTATTGTTAGATTTCCTAATATTACAAACTTGTGCCCTTTTGATGAAGTT<br>TTTAACGCCACCAGATTTGCATCTGTTTATGCTTGGAACAGGAAGAGAATCA<br>GCAACTGTGTTGCTGATTATTCTGTCCTATATAATTTCGCACCATTTTTCGCTT<br>TTAAGTGTTATGGAGTGTCTCCTACTAAATTAAATGATCTCTGCTTTACTAAT<br>GTCTATGCAGATTCATTTGTAATTAGAGGTAATGAAGTCAGCCAAATCGCTC<br>CAGGGCAAACTGGAAATATTGCTGATTATAATTATAAATTACCAGATGATTTT<br>ACAGGCTGCGTTATAGCTTGGAATTCTAACAAGCTTGATTCTAAGGTTGGTG<br>GTAATTATAATTACCGGTATAGATTGTTTAGGAAGTCTAATCTCAAACCTTTT<br>GAGAGAGATATTTCAACTGAAATCTATCAGGCCGGTAACAAACCTTGTAATG<br>GTGTTGCAGGTGTTAATTGTTACTTTCCTTTACAATCATATGGTTTCCGACCC<br>ACTTATGGTGTTGGTCACCAACCATACAGAGTAGTAGTACTTTCTTTTGAAC<br>TTCTACATGCACCAGCAACTGTTTGTGGACCTAAAAAGTCTACTAATTTGGT<br>TAAAAACAAATGTGTCAATTTCAACTTCAATGGTTTAACAGGCACAGGTGTT<br>CTTACTGAGTCTAACAAAAAGTTTCTGCCTTTCCAACAATTTGGCAGAGACA<br>TTGCTGACACTACTGATGCTGTCCGTGATCCACAGACACTTGAGATTCTTGA<br>CATTACACCATGTTCTTTTGGTGGTGTCAGTGTTATAACACCAGGAACAAAT<br>ACTTCTAACCAGGTTGCTGTTCTTTATCAGGGTGTTAACTGCACAGAAGTCC<br>CTGTTGCTATTCATGCAGATCAACTTACTCCTACTTGGCGTGTTTATTCTACA<br>GGTTCTAATGTTTTTCAAACACGTGCAGGCTGTTTAATAGGGGCTGAATATG<br>TCAACAACTCATATGAGTGTGACATACCCATTGGTGCAGGTATATGCGCTAGT<br>TATCAGACTCAGACTAAGTCTCATCGGCGGGCACGTAGTGTAGCTAGTCAAT<br>CCATCATTGCCTACACTATGTCACTTGGTGCAGAAAATTCAGTT
Omicron<br>BF.7<br>S gene	TCTTCAGGTTGGACAGCTGGTGCTGCAGCTTATTATGTGGGTTATCTTCAAC<br>CTAGGACTTTTCTATTAAAATATAATGAAAATGGAACCATTACAGATGCTGTA<br>GACTGTGCACTTGACCCTCTCTCAGAAACAAAGTGTACGTTGAAATCCTTC<br>ACTGTAGAAAAAGGAATCTATCAAACTTCTAACTTTAGAGTCCAACCAACA<br>GAATCTATTGTTAGATTTCCTAATATTACAAACTTGTGCCCTTTTGATGAAGTT<br>TTTAACGCCACCACATTTGCATCTGTTTATGCTTGGAACAGGAAGAGAATCA<br>GCAACTGTGTTGCTGATTATTCTGTCCTATATAATTTCGCACCATTTTTCGCTT<br>TTAAGTGTTATGGAGTGTCTCCTACTAAATTAAATGATCTCTGCTTTACTAAT<br>GTCTATGCAGATTCATTTGTAATTAGAGGTAATGAAGTCAGCCAAATCGCTC<br>CAGGGCAAACTGGAAATATTGCTGATTATAATTATAAATTACCAGATGATTTT<br>ACAGGCTGCGTTATAGCTTGGAATTCTAACAAGCTTGATTCTAAGGTTGGTG<br>GTAATTATAATTACCGGTATAGATTGTTTAGGAAGTCTAATCTCAAACCTTTT<br>GAGAGAGATATTTCAACTGAAATCTATCAGGCCGGTAACAAACCTTGTAATG<br>GTGTTGCAGGTGTTAATTGTTACTTTCCTTTACAATCATATGGTTTCCGACCC<br>ACTTATGGTGTTGGTCACCAACCATACAGAGTAGTAGTACTTTCTTTTGAAC<br>TTCTACATGCACCAGCAACTGTTTGTGGACCTAAAAAGTCTACTAATTTGGT<br>TAAAAACAAATGTGTCAATTTCAACTTCAATGGTTTAACAGGCACAGGTGTT<br>CTTACTGAGTCTAACAAAAAGTTTCTGCCTTTCCAACAATTTGGCAGAGACA<br>TTGCTGACACTACTGATGCTGTCCGTGATCCACAGACACTTGAGATTCTTGA<br>CATTACACCATGTTCTTTTGGTGGTGTCAGTGTTATAACACCAGGAACAAAT<br>ACTTCTAACCAGGTTGCTGTTCTTTATCAGGGTGTTAACTGCACAGAAGTCC

	CTGTTGCTATTCATGCAGATCAACTTACTCCTACTTGGCGTGTTTATTCTACA G

Note: The gene fragments of SARS-CoV-2 spike protein was partly inserted into the vector pUC57.

Table S2. Summary of the crRNAs and RPA primers sequences based on the target sites of SARS-COV-2 S gene in our study

Mutations of Spike protein	RPA primers	Sequences (5’→3’)
R346T	BF1	TGTTAGATTTCCTAATATTACAAACTTGTGCC
	BF2	TGTGCCCTTTTGATGAAGTTTTTAACGCCACC
	BR1	GTTAGAATTCCAAGCTATAACGCAGCCTGTAA
	BR2	GGCCTGATAGATTTCAGTTGAAATATCTCTCT
F486V;49X	OF1	CCGGTAACAAACCTTGTAATGGTGTTGCAGGT
	OF2	TTGAGAGAGATATTTCAACTGAAATCTATCAG
	OR1	ATCACGGACAGCATCAGTAGTGTCAGCAATGTC
	OR2	CATGTAGAAGTTCAAAAGAAAGTACTACTACTCTG

Mutations of Spike protein	crRNA	Sequences (5’→3’)
R346T	crRNA-346T-1	AAUUUCUACUAAGUGUAGAUACGCCACCACAUUUGCAUCU
	crRNA-346T-2	AAUUUCUACUAAGUGUAGAUACGCCAGCACAUUUGCAUCU
	crRNA-346T-3	AAUUUCUACUAAGUGUAGAUACGCCACGACAUUUGCAUCU
	crRNA-346T-4	AAUUUCUACUAAGUGUAGAUACGCCACCUCAUUUGCAUCU
F486V	crRNA-486V-1	AAUUUCUACUAAGUGUAGAUCAGGUGUUAAUUGUUACUUU
	crRNA-486V-2	AAUUUCUACUAAGUGUAGAUAGGUGUUAAUUGUUACUUUC
	crRNA-486V-3	AAUUUCUACUAAGUGUAGAUCAGCUGUUAAUUGUUACUUU
	crRNA-486V-4	AAUUUCUACUAAGUGUAGAUGUAAUGGUGUUGCAGGUGUU
49X	crRNA-49X	AAUUUCUACUAAGUGUAGAUCGAUCAUAUAGUUUCCGACC

Table S3. Summary of the p-RNAs sequences complementary to corresponding selected crRNAs based on "R5-3PC" principle.

p-RNAs	Sequences (5'→3')
346T-1	AGAUGCPclinkerAAAUGUPclinkerGGUGGCPclinkerGUAUCUA
486V-2	GAAAGUPclinkerAACAAUPclinkerUAACACPclinkerCUAUCUA
49X	GGUCGGPclinkerAAACUAPclinkerUAUGAUPclinkerCGAUCUA

Table S4. Comparison of the buffer composition

NEBuffer 2.1	10mM Tris-HCl(pH7.9), 50mM NaCl, 10mM $MgCl_2$, and 100ug/mL BSA
RPA reaction buffer	50mM Tris-HOAc (pH=7.9), 14mM MgOAc, 5% PEG20K, and 2mM DTT.

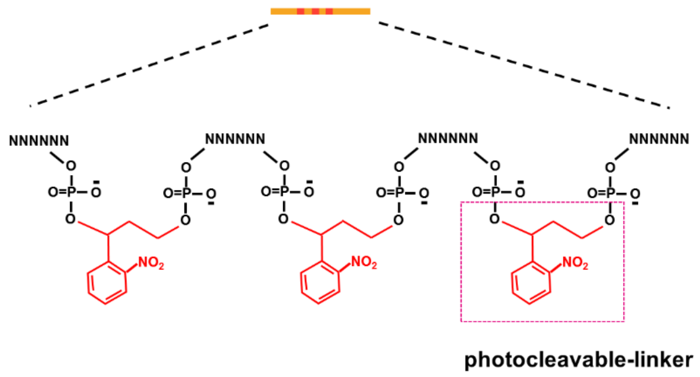

Figure S1. structural diagram of photocleavable RNA(p-RNA)

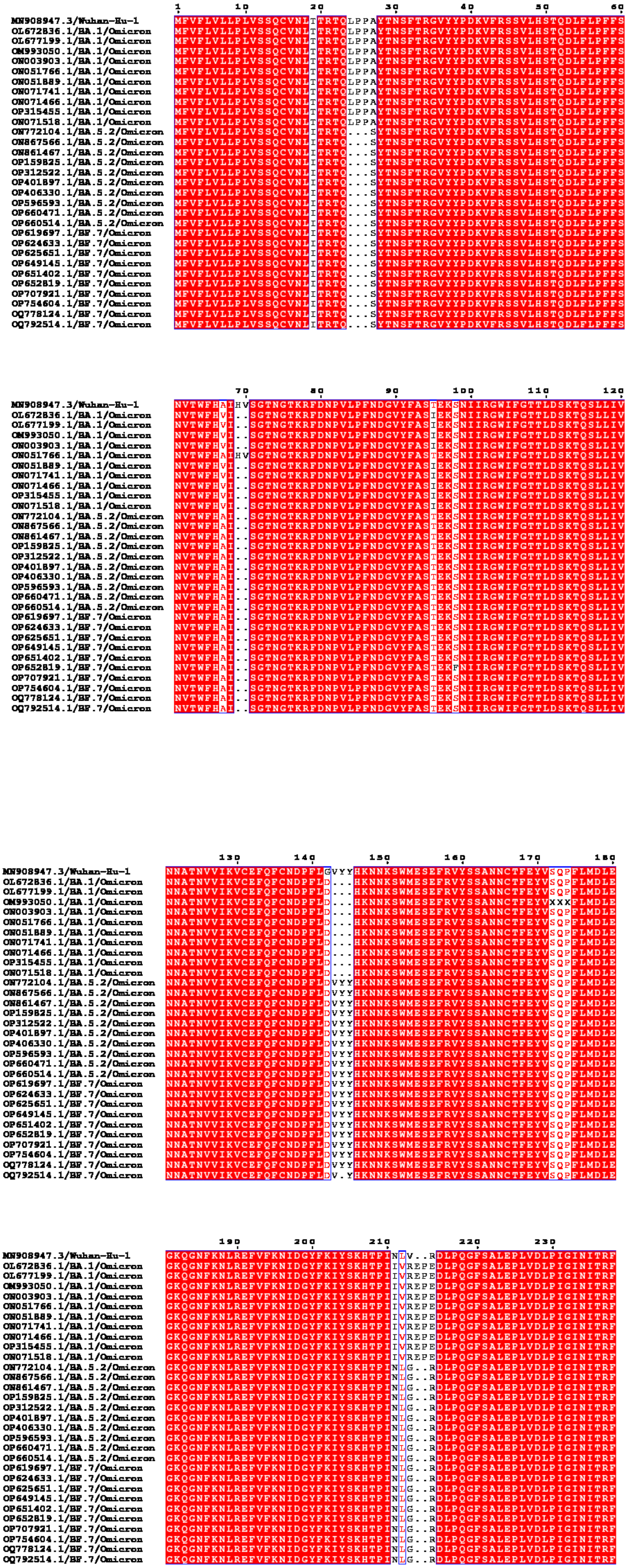

```
                                 240       250       260       270       280       290
MN908947.3/Wuhan-Hu-1        QTLLALHRSYLTPGDSSSGWTAGAAAYYVGYLQPRTFLLKYNENGTITDAVDCALDPLSE
OL672836.1/BA.1/Omicron      QTLLALHRSYLTPGDSSSGWTAGAAAYYVGYLQPRTFLLKYNENGTITDAVDCALDPLSE
OL677199.1/BA.1/Omicron      QTLLALHRSYLTPGDSSSGWTAGAAAYYVGYLQPRTFLLKYNENGTITDAVDCALDPLSE
OM993050.1/BA.1/Omicron      QTLLALHRSYLTPGDSSSGWTAGAAAYYVGYLQPRTFLLKYNENGTITDAVDCALDPLSE
ON003903.1/BA.1/Omicron      QTLLALHRSYLTPGDSSSGWTAGAAAYYVGYLQPRTFLLKYNENGTITDAVDCALDPLSE
ON051766.1/BA.1/Omicron      QTLLALHRSYLTPGDSSSGWTAGAAAYYVGYLQPRTFLLKYNENGTITDVVDCALDPLSE
ON051889.1/BA.1/Omicron      QTLLALHRSYLTPGDSSSGWTAGAAAYYVGYLQPRTFLLKYNENGTITDAVDCALDPLSE
ON071741.1/BA.1/Omicron      QTLLALHRSYLTPGDSSSGWTAGAAAYYVGYLQPRTFLLKYNENGTITDAVDCALDPLSE
ON071466.1/BA.1/Omicron      QTLLALHRSYLTPGDSSSGWTAGAAAYYVGYLQPRTFLLKYNENGTITDAVDCALDPLSE
OP315455.1/BA.1/Omicron      QTLLALHRSYLTPGDSSSGWTAGAAAYYVGYLQPRTFLLKYNENGTITDAVDCALDPLSE
ON071518.1/BA.1/Omicron      QTLLALHRSYLTPGDSSSGWTAGAAAYYVGYLQPRTFLLKYNENGTITDAVDCALDPLSE
ON772104.1/BA.5.2/Omicron    QTLLALHRSYLTPGDSSSGWTAGAAAYYVGYLQPRTFLLKYNENGTITDAVDCALDPLSE
ON867566.1/BA.5.2/Omicron    QTLLALHRSYLTPGDSSSGWTAGAAAYYVGYLQPRTFLLKYNENGTITDAVDCALDPLSE
ON861467.1/BA.5.2/Omicron    QTLLALHRSYLTPGDSSSGWTAGAAAYYVGYLQPRTFLLKYNENGTITDAVDCALDPLSE
OP159825.1/BA.5.2/Omicron    QTLLALHRSYLTPGDSSSGWTAGAAAYYVGYLQPRTFLLKYNENGTITDAVDCALDPLSE
OP312522.1/BA.5.2/Omicron    QTLLALHRSYLTPGDSSSGWTAGAAAYYVGYLQPRTFLLKYNENGTITDAVDCALDPLSE
OP401897.1/BA.5.2/Omicron    QTLLALHRSYLTPGDSSSGWTAGAAAYYVGYLQPRTFLLKYNENGTITDAVDCALDPLSE
OP406330.1/BA.5.2/Omicron    QTLLALHRSYLTPGDSSSGWTAGAAAYYVGYLQPRTFLLKYNENGTITDAVDCALDPLSE
OP596593.1/BA.5.2/Omicron    QTLLALHRSYLTPGDSSSGWTAGAAAYYVGYLQPRTFLLKYNENGTITDAVDCALDPLSE
OP660471.1/BA.5.2/Omicron    QTLLALHRSYLTPGDSSSGWTAGAAAYYVGYLQPRTFLLKYNENGTITDAVDCALDPLSE
OP660514.1/BA.5.2/Omicron    QTLLALHRSYLTPGDSSSGWTAGAAAYYVGYLQPRTFLLKYNENGTITDAVDCALDPLSE
OP619697.1/BF.7/Omicron      QTLLALHRSYLTPGDSSSGWTAGAAAYYVGYLQPRTFLLKYNENGTITDAVDCALDPLSE
OP624633.1/BF.7/Omicron      QTLLALHRSYLTPGDSSSGWTAGAAAYYVGYLQPRTFLLKYNENGTITDAVDCALDPLSE
OP625651.1/BF.7/Omicron      QTLLALHRSYLTPGDSSSGWTAGAAAYYVGYLQPRTFLLKYNENGTITDAVDCALDPLSE
OP649145.1/BF.7/Omicron      QTLLALHRSYLTPGDSSSGWTAGAAAYYVGYLQPRTFLLKYNENGTITDAVDCALDPLSE
OP651402.1/BF.7/Omicron      QTLLALHRSYLTPGDSFSGWTAGAAAYYVGYLQPRTFLLKYNENGTITDAVDCALDPLSE
OP652819.1/BF.7/Omicron      QTLLALHRSYLTPGDSSSGWTAGAAAYYVGYLQPRTFLLKYNENGTITDAVDCALDPLSE
OP707921.1/BF.7/Omicron      QTLLALHRSYLTPGDSSSGWTAGAAAYYVGYLQPRTFLLKYNENGTITDAVDCALDPLSE
OP754604.1/BF.7/Omicron      QTLLALHRSYLTPGDSSSGWTAGAAAYYVGYLQPRTFLLKYNENGTITDAVDCALDPLSE
OQ778124.1/BF.7/Omicron      QTLLALHRSYLTPGDSSSGWTAGAAAYYVGYLQPRTFLLKYNENGTITDAVDCALDPLSE
OQ792514.1/BF.7/Omicron      QTLLALHRSYLTPGDSSSGWTAGAAAYYVGYLQPRTFLLKYNENGTITDAVDCALDPLSE
```

```
                                 300       310       320       330       340       350
MN908947.3/Wuhan-Hu-1        TKCTLKSFTVEKGIYQTSNFRVQPTESIVRFPNITNLCPFGEVFNATRFASVYAWNRKRI
OL672836.1/BA.1/Omicron      TKCTLKSFTVEKGIYQTSNFRVQPTESIVRFPNITNLCPFDEVFNATRFASVYAWNRKRI
OL677199.1/BA.1/Omicron      TKCTLKSFTVEKGIYQTSNFRVQPTESIVRFPNITNLCPFDEVFNATRFASVYAWNRKRI
OM993050.1/BA.1/Omicron      TKCTLKSFTVEKGIYQTSNFRVQPTESIVRFPNITNLCPFDEVFNATRFASVYAWNRKRI
ON003903.1/BA.1/Omicron      TKCTLKSFTVEKGIYQTSNFRVQPTESIVRFPNITNLCPFDEVFNATRFASVYAWNRKRI
ON051766.1/BA.1/Omicron      TKCTLKSFTVEKGIYQTSNFRVQPTESIVRFPNITNLCPFDEVFNATKFASVYAWNRKRI
ON051889.1/BA.1/Omicron      TKCTLKSFTVEKGIYQTSNFRVQPTESIVRFPNITNLCPFDEVFNATKFASVYAWNRKRI
ON071741.1/BA.1/Omicron      TKCTLKSFTVEKGIYQTSNFRVQPTESIVRFPNITNLCPFDEVFNATKFASVYAWNRKRI
ON071466.1/BA.1/Omicron      TKCTLKSFTVEKGIYQTSNFRVQPTESIVRFPNITNLCPFDEVFNATRFASVYAWNRKRI
OP315455.1/BA.1/Omicron      TKCTLKSFTVEKGIYQTSNFRVQPTESIVRFPNITNLCPFDEVFNATRFASVYAWNRKRI
ON071518.1/BA.1/Omicron      TKCTLKSFTVEKGIYQTSNFRVQPTESIVRFPNITNLCPFDEVFNATKFASVYAWNRKRI
ON772104.1/BA.5.2/Omicron    TKCTLKSFTVEKGIYQTSNFRVQPTESIVRFPNITNLCPFDEVFNATRFASVYAWNRKRI
ON867566.1/BA.5.2/Omicron    TKCTLKSFTVEKGIYQTSNFRVQPTESIVRFPNITNLCPFDEVFNATRFASVYAWNRKRI
ON861467.1/BA.5.2/Omicron    TKCTLKSFTVEKGIYQTSNFRVQPTESIVRFPNITNLCPFDEVFNATRFASVYAWNRKRI
OP159825.1/BA.5.2/Omicron    TKCTLKSFTVEKGIYQTSNFRVQPTESIVRFPNITNLCPFDEVFNATRFASVYAWNRKRI
OP312522.1/BA.5.2/Omicron    TKCTLKSFTVEKGIYQTSNFRVQPTESIVRFPNITNLCPFDEVFNATRFASVYAWNRKRI
OP401897.1/BA.5.2/Omicron    TKCTLKSFTVEKGIYQTSNFRVQPTESIVRFPNITNLCPFDEVFNATRFASVYAWNRKRI
OP406330.1/BA.5.2/Omicron    TKCTLKSFTVEKGIYQTSNFRVQPTESIVRFPNITNLCPFDEVFNATKFASVYAWNRKRI
OP596593.1/BA.5.2/Omicron    TKCTLKSFTVEKGIYQTSNFRVQPTESIVRFPNITNLCPFDEVFNATRFASVYAWNRKRI
OP660471.1/BA.5.2/Omicron    TKCTLKSFTVEKGIYQTSNFRVQPTESIVRFPNITNLCPFDEVFNATRFASVYAWNRKRI
OP660514.1/BA.5.2/Omicron    TKCTLKSFTVEKGIYQTSNFRVQPTESIVRFPNITNLCPFDEVFNATRFASVYAWNRKRI
OP619697.1/BF.7/Omicron      TKCTLKSFTVEKGIYQTSNFRVQPTESIVRFPNITNLCPFDEVFNATTFASVYAWNRKRI
OP624633.1/BF.7/Omicron      TKCTLKSFTVEKGIYQTSNFRVQPTESIVRFPNITNLCPFDEVFNATTFASVYAWNRKRI
OP625651.1/BF.7/Omicron      TKCTLKSFTVEKGIYQTSNFRVQPTESIVRFPNITNLCPFDEVFNATTFASVYAWNRKRI
OP649145.1/BF.7/Omicron      TKCTLKSFTVEKGIYQTSNFRVQPTESIVRFPNITNLCPFDEVFNATTFASVYAWNRKRI
OP651402.1/BF.7/Omicron      TKCTLKSFTVEKGIYQTSNFRVQPTESIVRFPNITNLCPFDEVFNATTFASVYAWNRKRI
OP652819.1/BF.7/Omicron      TKCTLKSFTVEKGIYQTSNFRVQPTESIVRFPNITNLCPFDEVFNATTFASVYAWNRKRI
OP707921.1/BF.7/Omicron      TKCTLKSFTVEKGIYQTSNFRVQPTESIVRFPNITNLCPFDEVFNATTFASVYAWNRKRI
OP754604.1/BF.7/Omicron      TKCTLKSFTVEKGIYQTSNFRVQPTESIVRFPNITNLCPFDEVFNATTFASVYAWNRKRI
OQ778124.1/BF.7/Omicron      TKCTLKSFTVEKGIYQTSNFRVQPTESIVRFPNITNLCPFDEVFNATTFASVYAWNRKRI
OQ792514.1/BF.7/Omicron      TKCTLKSFTVEKGIYQTSNFRVQPTESIVRFPNITNLCPFDEVFNATTFASVYAWNRKRI
```

```
                                 360       370       380       390       400       410
MN908947.3/Wuhan-Hu-1        SNCVADYSVLYNSASFSTFKCYGVSPTKLNDLCFTNVYADSFVIRGDEVRQIAPGQTGKI
OL672836.1/BA.1/Omicron      SNCVADYSVLYNLAPFFTFKCYGVSPTKLNDLCFTNVYADSFVIRGDEVRQIAPGQTGNI
OL677199.1/BA.1/Omicron      SNCVADYSVLYNLAPFFTFKCYGVSPTKLNDLCFTNVYADSFVIRGDEVRQIAPGQTGNI
OM993050.1/BA.1/Omicron      SNCVADYSVLYNLAPFFTFKCYGVSPTKLNDLCFTNVYADSFVIRGDEVRQIAPGQTGNI
ON003903.1/BA.1/Omicron      SNCVADYSVLYNLAPFFTFKCYGVSPTKLNDLCFTNVYADSFVIRGDEVRQIAPGQTGKI
ON051766.1/BA.1/Omicron      SNCVADYSVLYNLAPFFTFKCYGVSPTKLNDLCFTNVYADSFVIRGDEVRQIAPGQTGKI
ON051889.1/BA.1/Omicron      SNCVADYSVLYNLAPFFTFKCYGVSPTKLNDLCFTNVYADSFVIRGDEVRQIAPGQTGKI
ON071741.1/BA.1/Omicron      SNCVADYSVLYNLAPFFTFKCYGVSPTKLNDLCFTNVYADSFVIRGDEVRQIAPGQTGKI
ON071466.1/BA.1/Omicron      SNCVADYSVLYNLAPFFTFKCYGVSPTKLNDLCFTNVYADSFVIRGDEVRQIAPGQTGKI
OP315455.1/BA.1/Omicron      SNCVADYSVLYNLAPFFTFKCYGVSPTKLNDLCFTNVYADSFVIRGDEVRQIAPGQTGNI
ON071518.1/BA.1/Omicron      SNCVADYSVLYNLAPFFTFKCYGVSPTKLNDLCFTNVYADSFVIRGDEVRQIAPGQTGKI
ON772104.1/BA.5.2/Omicron    SNCVADYSVLYNFAPFFAFKCYGVSPTKLNDLCFTNVYADSFVIRGNEVSQIAPGQTGNI
ON867566.1/BA.5.2/Omicron    SNCVADYSVLYNFAPFFAFKCYGVSPTKLNDLCFTNVYADSFVIRGNEVSQIAPGQTGNI
ON861467.1/BA.5.2/Omicron    SNCVADYSVLYNFAPFFAFKCYGVSPTKLNDLCFTNVYADSFVIRGNEVSQIAPGQTGNI
OP159825.1/BA.5.2/Omicron    SNCVADYSVLYNFAPFFAFKCYGVSPTKLNDLCFTNVYADSFVIRGNEVSQIAPGQTGNI
OP312522.1/BA.5.2/Omicron    SNCVADYSVLYNFAPFFAFKCYGVSPTKLNDLCFTNVYADSFVIRGNEVSQIAPGQTGNI
OP401897.1/BA.5.2/Omicron    SNCVADYSVLYNFAPFFAFKCYGVSPTKLNDLCFTNVYADSFVIRGNEVSQIAPGQTGNI
OP406330.1/BA.5.2/Omicron    SNCVADYSVLYNFAPFFAFKCYGVSPTKLNDLCFTNVYADSFVIRGNEVSQIAPGQTGNI
OP596593.1/BA.5.2/Omicron    SNCVADYSVLYNFAPFFAFKCYGVSPTKLNDLCFTNVYADSFVIRGNEVSQIAPGQTGNI
OP660471.1/BA.5.2/Omicron    SNCVADYSVLYNFAPFFAFKCYGVSPTKLNDLCFTNVYADSFVIRGNEVSQIAPGQTGNI
OP660514.1/BA.5.2/Omicron    SNCVADYSVLYNFAPFFAFKCYGVSPTKLNDLCFTNVYADSFVIRGNEVSQIAPGQTGNI
OP619697.1/BF.7/Omicron      SNCVADYSVLYNFAPFFAFKCYGVSPTKLNDLCFTNVYADSFVIRGNEVSQIAPGQTGNI
OP624633.1/BF.7/Omicron      SNCVADYSVLYNFAPFFAFKCYGVSPTKLNDLCFTNVYADSFVIRGNEVSQIAPGQTGNI
OP625651.1/BF.7/Omicron      SNCVADYSVLYNFAPFFAFKCYGVSPTKLNDLCFTNVYADSFVIRGNEVSQIAPGQTGNI
OP649145.1/BF.7/Omicron      SNCVADYSVLYNFAPFFAFKCYGVSPTKLNDLCFTNVYADSFVIRGNEVSQIAPGQTGNI
OP651402.1/BF.7/Omicron      SNCVADYSVLYNFAPFFAFKCYGVSPTKLNDLCFTNVYADSFVIRGNEVSQIAPGQTGNI
OP652819.1/BF.7/Omicron      SNCVADYSVLYNFAPFFAFKCYGVSPTKLNDLCFTNVYADSFVIRGNEVSQIAPGQTGNI
OP707921.1/BF.7/Omicron      SNCVADYSVLYNFAPFFAFKCYGVSPTKLNDLCFTNVYADSFVIRGNEVSQIAPGQTGNI
OP754604.1/BF.7/Omicron      SNCVADYSVLYNFAPFFAFKCYGVSPTKLNDLCFTNVYADSFVIRGNEVSQIAPGQTGNI
OQ778124.1/BF.7/Omicron      SNCVADYSVLYNFAPFFAFKCYGVSPTKLNDLCFTNVYADSFVIRGNEVSQIAPGQTGNI
OQ792514.1/BF.7/Omicron      SNCVADYSVLYNFAPFFAFKCYGVSPTKLNDLCFTNVYADSFVIRGNEVSQIAPGQTGNI
```

```
                                 420       430       440       450       460       470
MN908947.3/Wuhan-Hu-1        ADYNYKLPDDFTGCVIAWNSNNLDSKVGGNYNYLYRLFRKSNLKPFERDISTEIYQAGST
OL672836.1/BA.1/Omicron      ADYNYKLPDDFTGCVIAWNSNKLDSKVSGNYNYLYRLFRKSNLKPFERDISTEIYQAGNK
OL677199.1/BA.1/Omicron      ADYNYKLPDDFTGCVIAWNSNKLDSKVSGNYNYLYRLFRKSNLKPFERDISTEIYQAGNK
OM993050.1/BA.1/Omicron      ADYNYKLPDDFTGCVIAWNSNKLDSKVSGNYNYLYRLFRKSNLKPFERDISTEIYQAGNK
ON003903.1/BA.1/Omicron      ADYNYKLPDDFTGCVIAWNSNNLDSKVGGNYNYLYRLFRKSNLKPFERDISTEIYQAGNT
ON051766.1/BA.1/Omicron      ADYNYKLPDDFTGCVIAWNSNNLDSKVGGNYNYLYRLFRKSNLKPFERDISTEIYQAGNK
ON051889.1/BA.1/Omicron      ADYNYKLPDDFTGCVIAWNSNNLDSKVGGNYNYLYRLFRKSNLKPFERDISTEIYQAGNT
ON071741.1/BA.1/Omicron      ADYNYKLPDDFTGCVIAWNSNNLDSKVGGNYNYLYRLFRKSNLKPFERDISTEIYQAGNK
ON071466.1/BA.1/Omicron      ADYNYKLPDDFTGCVIAWNSNNLDSKVGGNYNYLYRLFRKSNLKPFERDISTEIYQAGST
OP315455.1/BA.1/Omicron      ADYNYKLPDDFTGCVIAWNSNKLDSKVSGNYNYLYRLFRKSNLKPFERDISTEIYQAGNK
ON071518.1/BA.1/Omicron      ADYNYKLPDDFTGCVIAWNSNNLDSKVGGNYNYLYRLFRKSNLKPFERDISTEIYQAGNK
ON772104.1/BA.5.2/Omicron    ADYNYKLPDDFTGCVIAWNSNKLDSKVGGNYNYRYRLFRKSNLKPFERDISTEIYQAGNK
ON867566.1/BA.5.2/Omicron    ADYNYKLPDDFTGCVIAWNSNKLDSKVGGNYNYRYRLFRKSNLKPFERDISTEIYQAGNK
ON861467.1/BA.5.2/Omicron    ADYNYKLPDDFTGCVIAWNSNKLDSKVGGNYNYRYRLFRKSNLKPFERDISTEIYQAGNK
OP159825.1/BA.5.2/Omicron    ADYNYKLPDDFTGCVIAWNSNKLDSKVGGNYNYRYRLFRKSNLKPFERDISTEIYQAGNK
OP312522.1/BA.5.2/Omicron    ADYNYKLPDDFTGCVIAWNSNKLDSKVGGNYNYRYRLFRKSNLKPFERDISTEIYQAGNK
OP401897.1/BA.5.2/Omicron    ADYNYKLPDDFTGCVIAWNSNKLDSKVGGNYNYRYRLFRKSNLKPFERDISTEIYQAGNK
OP406330.1/BA.5.2/Omicron    ADYNYKLPDDFTGCVIAWNSNKLDSKVGGNYNYRYRLFRKSNLKPFERDISTEIYQAGNK
OP596593.1/BA.5.2/Omicron    ADYNYKLPDDFTGCVIAWNSNKLDSKVGGNYNYRYRLFRKSNLKPFERDISTEIYQAGNK
OP660471.1/BA.5.2/Omicron    ADYNYKLPDDFTGCVIAWNSNKLDSKVGGNYNYRYRLFRKSNLKPFERDISTEIYQAGNK
OP660514.1/BA.5.2/Omicron    ADYNYKLPDDFTGCVIAWNSNKLDSKVGGNYNYRYRLFRKSNLKPFERDISTEIYQAGNK
OP619697.1/BF.7/Omicron      ADYNYKLPDDFTGCVIAWNSNKLDSKVGGNYNYRYRLFRKSNLKPFERDISTEIYQAGNK
OP624633.1/BF.7/Omicron      ADYNYKLPDDFTGCVIAWNSNKLDSKVGGNYNYRYRLFRKSNLKPFERDISTEIYQAGNK
OP625651.1/BF.7/Omicron      ADYNYKLPDDFTGCVIAWNSNKLDSKVGGNYNYRYRLFRKSNLKPFERDISTEIYQAGNK
OP649145.1/BF.7/Omicron      ADYNYKLPDDFTGCVIAWNSNKLDSKVGGNYNYRYRLFRKSNLKPFERDISTEIYQAGNK
OP651402.1/BF.7/Omicron      ADYNYKLPDDFTGCVIAWNSNKLDSKVGGNYNYRYRLFRKSNLKPFERDISTEIYQAGNK
OP652819.1/BF.7/Omicron      ADYNYKLPDDFTGCVIAWNSNKLDSKVGGNYNYRYRLFRKSNLKPFERDISTEIYQAGNK
OP707921.1/BF.7/Omicron      ADYNYKLPDDFTGCVIAWNSNKLDSKVGGNYNYRYRLFRKSNLKPFERDISTEIYQAGNK
OP754604.1/BF.7/Omicron      ADYNYKLPDDFTGCVIAWNSNKLDSKVGGNYNYRYRLFRKSNLKPFERDISTEIYQAGNK
OQ778124.1/BF.7/Omicron      ADYNYKLPDDFTGCVIAWNSNKLDSKVGGNYNYRYRLFRKSNLKPFERDISTEIYQAGNK
OQ792514.1/BF.7/Omicron      ADYNYKLPDDFTGCVIAWNSNKLDSKVGGNYNYRYRLFRKSNLKPFERDISTEIYQAGNK
```

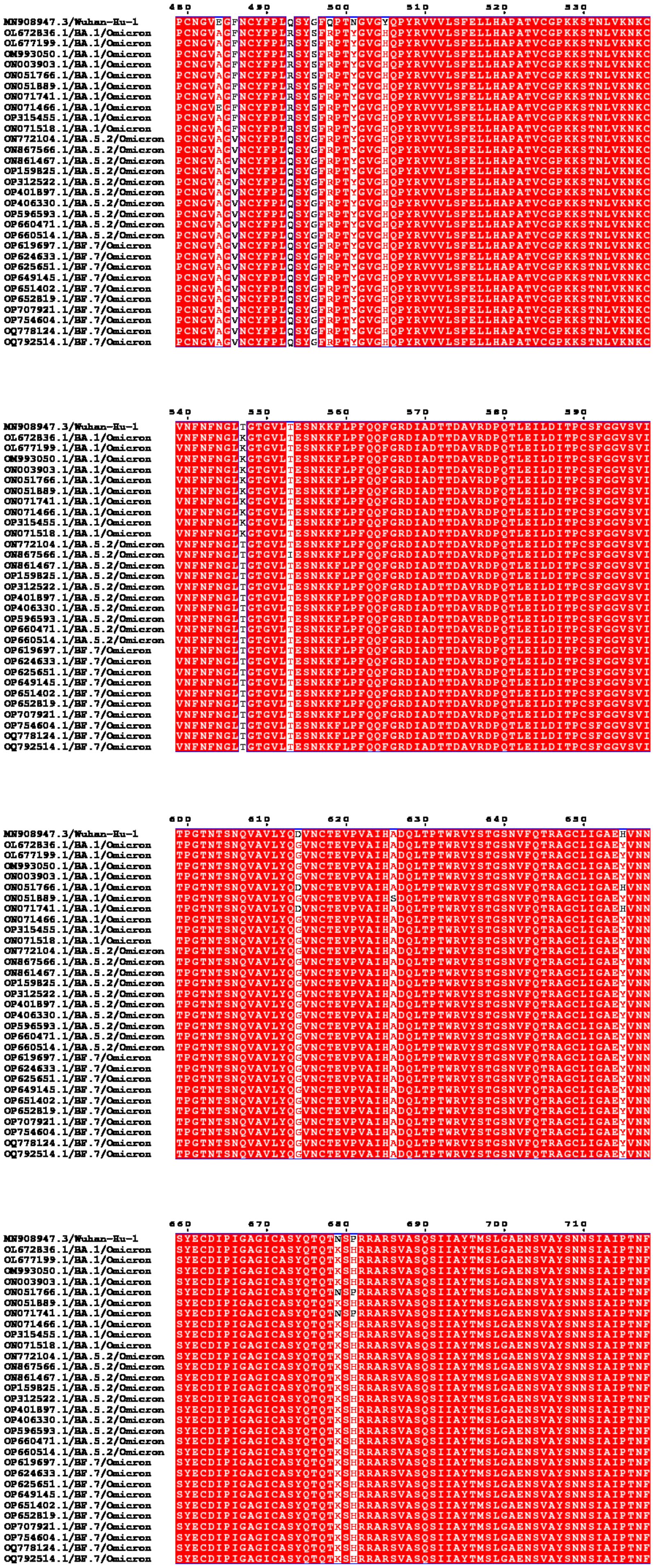

```
                           720       730       740       750       760       770
MN908947.3/Wuhan-Hu-1      TISVTTEILPVSMTKTSVDCTMYICGDSTECSNLLLQYGSFCTQLNRALTGIAVEQDKNT
OL672836.1/BA.1/Omicron    TISVTTEILPVSMTKTSVDCTMYICGDSTECSNLLLQYGSFCTQLKRALTGIAVEQDKNT
OL677199.1/BA.1/Omicron    TISVTTEILPVSMTKTSVDCTMYICGDSTECSNLLLQYGSFCTQLKRALTGIAVEQDKNT
OM993050.1/BA.1/Omicron    TISVTTEILPVSMTKTSVDCTMYICGDSTECSNLLLQYGSFCTQLKRALTGIAVEQDKNT
ON003903.1/BA.1/Omicron    TISVTTEILPVSMTKTSVDCTMYICGDSTECSNLLLQYGSFCTQLNRALTGIAVEQDKNT
ON051766.1/BA.1/Omicron    TISVTTEILPVSMTKTSVDCTMYICGDSTECSNLLLQYGSFCTQLNRALTGIAVEQDKNT
ON051889.1/BA.1/Omicron    TISVTTEILPVSMTKTSVDCTMYICGDSTECSNLLLQYGSFCTQLNRALTGIAVEQDKNT
ON071741.1/BA.1/Omicron    TISVTTEILPVSMTKTSVDCTMYICGDSTECSNLLLQYGSFCTQLNRALTGIAVEQDKNT
ON071466.1/BA.1/Omicron    TISVTTEILPVSMTKTSVDCTMYICGDSTECSNLLLQYGSFCTQLNRALTGIAVEQDKNT
OP315455.1/BA.1/Omicron    TISVTTEILPVSMTKTSVDCTMYICGDSTECSNLLLQYGSFCTQLKRALTGIAVEQDKNT
ON071518.1/BA.1/Omicron    TISVTTEILPVSMTKTSVDCTMYICGDSTECSNLLLQYGSFCTQLNRALTGIAVEQDKNT
ON772104.1/BA.5.2/Omicron  TISVTTEILPVSMTKTSVDCTMYICGDSTECSNLLLQYGSFCTQLKRALTGIAVEQDKNT
ON867566.1/BA.5.2/Omicron  TISVTTEILPVSMTKTSVDCTMYICGDSTECSNLLLQYGSFCTQLKRALTGIAVEQDKNT
ON861467.1/BA.5.2/Omicron  TISVTTEILPVSMTKTSVDCTMYICGDSTECSNLLLQYGSFCTQLKRALTGIAVEQDKNT
OP159825.1/BA.5.2/Omicron  TISVTTEILPVSMTKTSVDCTMYICGDSTECSNLLLQYGSFCTQLKRALTGIAVEQDKNT
OP312522.1/BA.5.2/Omicron  TISVTTEILPVSMTKTSVDCTMYICGDSTECSNLLLQYGSFCTQLKRALTGIAVEQDKNT
OP401897.1/BA.5.2/Omicron  TISVTTEILPVSMTKTSVDCTMYICGDSTECSNLLLQYGSFCTQLKRALTGIAVEQDKNT
OP406330.1/BA.5.2/Omicron  TISVTTEILPVSMTKTSVDCTMYICGDSTECSNLLLQYGSFCTQLKRALTGIAVEQDKNT
OP596593.1/BA.5.2/Omicron  TISVTTEILPVSMTKTSVDCTMYICGDSTECSNLLLQYGSFCTQLKRALTGIAVEQDKNT
OP660471.1/BA.5.2/Omicron  TISVTTEILPVSMTKTSVDCTMYICGDSTECSNLLLQYGSFCTQLKRALTGIAVEQDKNT
OP660514.1/BA.5.2/Omicron  TISVTTEILPVSMTKTSVDCTMYICGDSTECSNLLLQYGSFCTQLKRALTGIAVEQDKNT
OP619697.1/BF.7/Omicron    TISVTTEILPVSMTKTSVDCTMYICGDSTECSNLLLQYGSFCTQLKRALTGIAVEQDKNT
OP624633.1/BF.7/Omicron    TISVTTEILPVSMTKTSVDCTMYICGDSTECSNLLLQYGSFCTQLKRALTGIAVEQDKNT
OP625651.1/BF.7/Omicron    TISVTTEILPVSMTKTSVDCTMYICGDSTECSNLLLQYGSFCTQLKRALTGIAVEQDKNT
OP649145.1/BF.7/Omicron    TISVTTEILPVSMTKTSVDCTMYICGDSTECSNLLLQYGSFCTQLKRALTGIAVEQDKNT
OP651402.1/BF.7/Omicron    TISVTTEILPVSMTKTSVDCTMYICGDSTECSNLLLQYGSFCTQLKRALTGIAVEQDKNT
OP652819.1/BF.7/Omicron    TISVTTEILPVSMTKTSVDCTMYICGDSTECSNLLLQYGSFCTQLKRALTGIAVEQDKNT
OP707921.1/BF.7/Omicron    TISVTTEILPVSMTKTSVDCTMYICGDSTECSNLLLQYGSFCTQLKRALTGIAVEQDKNT
OP754604.1/BF.7/Omicron    TISVTTEILPVSMTKTSVDCTMYICGDSTECSNLLLQYGSFCTQLKRALTGIAVEQDKNT
OQ778124.1/BF.7/Omicron    TISVTTEILPVSMTKTSVDCTMYICGDSTECSNLLLQYGSFCTQLKRALTGIAVEQDKNT
OQ792514.1/BF.7/Omicron    TISVTTEILPVSMTKTSVDCTMYICGDSTECSNLLLQYGSFCTQLKRALTGIAVEQDKNT
```

```
                           780       790       800       810       820       830
MN908947.3/Wuhan-Hu-1      QEVFAQVKQIYKTPPIKDFGGFNFSQILPDPSKPSKRSFIEDLLFNKVTLADAGFIKQYG
OL672836.1/BA.1/Omicron    QEVFAQVKQIYKTPPIKYFGGFNFSQILPDPSKPSKRSFIEDLLFNKVTLADAGFIKQYG
OL677199.1/BA.1/Omicron    QEVFAQVKQIYKTPPIKYFGGFNFSQILPDPSKPSKRSFIEDLLFNKVTLADAGFIKQYG
OM993050.1/BA.1/Omicron    QEVFAQVKQIYKTPPIKYFGGFNFSQILPDPSKPSKRSFIEDLLFNKVTLADAGFIKQYG
ON003903.1/BA.1/Omicron    QEVFAQVKQIYKTPPIKYFGGFNFSQILPDPSKPSKRSFIEDLLFNKVTLADAGFIKQYG
ON051766.1/BA.1/Omicron    QEVFAQVKQIYKTPPIKYFGGFNFSQILPDPSKPSKRSFIEDLLFNKVTLADAGFIKQYG
ON051889.1/BA.1/Omicron    QEVFAQVKQIYKTPPIKYFGGFNFSQILPDPSKPSKRSFIEDLLFNKVTLADAGFIKQYG
ON071741.1/BA.1/Omicron    QEVFAQVKQIYKTPPIKYFGGFNFSQILPDPSKPSKRSFIEDLLFNKVTLADAGFIKQYG
ON071466.1/BA.1/Omicron    QEVFAQVKQIYKTPPIKDFGGFNFSQILPDPSKPSKRSFIEDLLFNKVTLADAGFIKQYG
OP315455.1/BA.1/Omicron    QEVFAQVKQIYKTPPIKYFGGFNFSQILPDPSKPSKRSFIEDLLFNKVTLADAGFIKQYG
ON071518.1/BA.1/Omicron    QEVFAQVKQIYKTPPIKYFGGFNFSQILPDPSKPSKRSFIEDLLFNKVTLADAGFIKQYG
ON772104.1/BA.5.2/Omicron  QEVFAQVKQIYKTPPIKYFGGFNFSQILPDPSKPSKRSFIEDLLFNKVTLADAGFIKQYG
ON867566.1/BA.5.2/Omicron  QEVFAQVKQIYKTPPIKYFGGFNFSQILPDPSKPSKRSFIEDLLFNKVTLADAGFIKQYG
ON861467.1/BA.5.2/Omicron  QEVFAQVKQIYKTPPIKYFGGFNFSQILPDPSKPSKRSFIEDLLFNKVTLADAGFIKQYG
OP159825.1/BA.5.2/Omicron  QEVFAQVKQIYKTPPIKYFGGFNFSQILPDPSKPSKRSFIEDLFFNKVTLADAGFIKQYG
OP312522.1/BA.5.2/Omicron  QEVFAQVKQIYKTPPIKYFGGFNFSQILPDPSKPSKRSFIEDLLFNKVTLADAGFIKQYG
OP401897.1/BA.5.2/Omicron  QEVFAQVKQIYKTPPIKYFGGFNFSQILPDPSKPSKRSFIEDLLFNKVTLADAGFIKQYG
OP406330.1/BA.5.2/Omicron  QEVFAQVKQIYKTPPIKYFGGFNFSQILPDPSKPSKRSFIEDLLFNKVTLADAGFIKQYG
OP596593.1/BA.5.2/Omicron  QEVFAQVKQIYKTPPIKYFGGFNFSQILPDPSKPSKRSFIEDLLFNKVTLADAGFIKQYG
OP660471.1/BA.5.2/Omicron  QEVFAQVKQIYKTPPIKYFGGFNFSQILPDPSKPSKRSFIEDLLFNKVTLADAGFIKQYG
OP660514.1/BA.5.2/Omicron  QEVFAQVKQIYKTPPIKYFGGFNFSQILPDPSKPSKRSFIEDLLFNKVTLADAGFIKQYG
OP619697.1/BF.7/Omicron    QEVFAQVKQIYKTPPIKYFGGFNFSQILPDPSKPSKRSFIEDLLFNKVTLADAGFIKQYG
OP624633.1/BF.7/Omicron    QEVFAQVKQIYKTPPIKYFGGFNFSQILPDPSKPSKRSFIEDLLFNKVTLADAGFIKQYG
OP625651.1/BF.7/Omicron    QEVFAQVKQIYKTPPIKYFGGFNFSQILPDPSKPSKRSFIEDLLFNKVTLADAGFIKQYG
OP649145.1/BF.7/Omicron    QEVFAQVKQIYKTPPIKYFGGFNFSQILPDPSKPSKRSFIEDLLFNKVTLADAGFIKQYG
OP651402.1/BF.7/Omicron    QEVFAQVKQIYKTPPIKYFGGFNFSQILPDPSKPSKRSFIEDLLFNKVTLADAGFIKQYG
OP652819.1/BF.7/Omicron    QEVFAQVKQIYKTPPIKYFGGFNFSQILPDPSKPSKRSFIEDLLFNKVTLADAGFIKQYG
OP707921.1/BF.7/Omicron    QEVFAQVKQIYKTPPIKYFGGFNFSQILPDPSKPSKRSFIEDLLFNKVTLADAGFIKQYG
OP754604.1/BF.7/Omicron    QEVFAQVKQIYKTPPIKYFGGFNFSQILPDPSKPSKRSFIEDLLFNKVTLADAGFIKQYG
OQ778124.1/BF.7/Omicron    QEVFAQVKQIYKTPPIKYFGGFNFSQILPDPSKPSKRSFIEDLLFNKVTLADAGFIKQYG
OQ792514.1/BF.7/Omicron    QEVFAQVKQIYKTPPIKYFGGFNFSQILPDPSKPSKRSFIEDLLFNKVTLADAGFIKQYG
```

```
                           840       850       860       870       880       890
MN908947.3/Wuhan-Hu-1      DCLGDIAARDLICAQKFNGLTVLPPLLTDEMIAQYTSALLAGTITSGWTFGAGAALQIPF
OL672836.1/BA.1/Omicron    DCLGDIAARDLICAQKFKGLTVLPPLLTDEMIAQYTSALLAGTITSGWTFGAGAALQIPF
OL677199.1/BA.1/Omicron    DCLGDIAARDLICAQKFKGLTVLPPLLTDEMIAQYTSALLAGTITSGWTFGAGAALQIPF
OM993050.1/BA.1/Omicron    DCLGDIAARDLICAQKFKGLTVLPPLLTDEMIAQYTSALLAGTITSGWTFGAGAALQIPF
ON003903.1/BA.1/Omicron    DCLGDIAARDLICAQKFKGLTVLPPLLTDEMIAQYTSALLAGTITSGWTFGAGAALQIPF
ON051766.1/BA.1/Omicron    DCLGDIAARDLICAQKFKGLTVLPPLLTDEMIAQYTSALLAGTITSGWTFGAGAALQIPF
ON051889.1/BA.1/Omicron    DCLGDIAARDLICAQKFKGLTVLPPLLTDEMIAQYTSALLAGTITSGWTFGAGAALQIPF
ON071741.1/BA.1/Omicron    DCLGDIAARDLICAQKFKGLTVLPPLLTDEMIAQYTSALLAGTITSGWTFGAGAALQIPF
ON071466.1/BA.1/Omicron    DCLGDIAARDLICAQKFNGLTVLPPLLTDEMIAQYTSALLAGTITSGWTFGAGAALQIPF
OP315455.1/BA.1/Omicron    DCLGDIAARDLICAQKFKGLTVLPPLLTDEMIAQYTSALLAGTITSGWTFGAGAALQIPF
ON071518.1/BA.1/Omicron    DCLGDIAARDLICAQKFKGLTVLPPLLTDEMIAQYTSALLAGTITSGWTFGAGAALQIPF
ON772104.1/BA.5.2/Omicron  DCLGDIAARDLICAQKFNGLTVLPPLLTDEMIAQYTSALLAGTITSGWTFGAGAALQIPF
ON867566.1/BA.5.2/Omicron  DCLGDIAARDLICAQKFNGLTVLPPLLTDEMIAQYTSALLAGTITSGWTFGAGAALQIPF
ON861467.1/BA.5.2/Omicron  DCLGDIAARDLICAQKFNGLTVLPPLLTDEMIAQYTSALLAGTITSGWTFGAGAALQIPF
OP159825.1/BA.5.2/Omicron  DCLGDIAARDLICAQKFNGLTVLPPLLTDEMIAQYTSALLAGTITSGWTFGAGAALQIPF
OP312522.1/BA.5.2/Omicron  DCLGDIAARDLICAQKFNGLTVLPPLLTDEMIAQYTSALLAGTITSGWTFGAGAALQIPF
OP401897.1/BA.5.2/Omicron  DCLGDIAARDLICAQKFNGLTVLPPLLTDEMIAQYTSALLAGTITSGWTFGAGAALQIPF
OP406330.1/BA.5.2/Omicron  DCLGDIAARDLICAQKFNGLTVLPPLLTDEMIAQYTSALLAGTITSGWTFGAGAALQIPF
OP596593.1/BA.5.2/Omicron  DCLGDIAARDLICAQKFNGLTVLPPLLTDEMIAQYTSALLAGTITSGWTFGAGAALQIPF
OP660471.1/BA.5.2/Omicron  DCLGDIAARDLICAQKFNGLTVLPPLLTDEMIAQYTSALLAGTITSGWTFGAGAALQIPF
OP660514.1/BA.5.2/Omicron  DCLGDIAARDLICAQKFNGLTVLPPLLTDEMIAQYTSALLAGTITSGWTFGAGAALQIPF
OP619697.1/BF.7/Omicron    DCLGDIAARDLICAQKFNGLTVLPPLLTDEMIAQYTSALLAGTITSGWTFGAGAALQIPF
OP624633.1/BF.7/Omicron    DCLGDIAARDLICAQKFNGLTVLPPLLTDEMIAQYTSALLAGTITSGWTFGAGAALQIPF
OP625651.1/BF.7/Omicron    DCLGDIAARDLICAQKFNGLTVLPPLLTDEMIAQYTSALLAGTITSGWTFGAGAALQIPF
OP649145.1/BF.7/Omicron    DCLGDIAARDLICAQKFNGLTVLPPLLTDEMIAQYTSALLAGTITSGWTFGAGAALQIPF
OP651402.1/BF.7/Omicron    DCLGDIAARDLICAQKFNGLTVLPPLLTDEMIAQYTSALLAGTITSGWTFGAGAALQIPF
OP652819.1/BF.7/Omicron    DCLGDIAARDLICAQKFNGLTVLPPLLTDEMIAQYTSALLAGTITSGWTFGAGAALQIPF
OP707921.1/BF.7/Omicron    DCLGDIAARDLICAQKFNGLTVLPPLLTDEMIAQYTSALLAGTITSGWTFGAGAALQIPF
OP754604.1/BF.7/Omicron    DCLGDIAARDLICAQKFNGLTVLPPLLTDEMIAQYTSALLAGTITSGWTFGAGAALQIPF
OQ778124.1/BF.7/Omicron    DCLGDIAARDLICAQKFNGLTVLPPLLTDEMIAQYTSALLAGTITSGWTFGAGAALQIPF
OQ792514.1/BF.7/Omicron    DCLGDIAARDLICAQKFNGLTVLPPLLTDEMIAQYTSALLAGTITSGWTFGAGAALQIPF
```

```
                           900       910       920       930       940       950
MN908947.3/Wuhan-Hu-1      AMQMAYRFNGIGVTQNVLYENQKLIANQFNSAIGKIQDSLSSTASALGKLQDVVNQNAQA
OL672836.1/BA.1/Omicron    AMQMAYRFNGIGVTQNVLYENQKLIANQFNSAIGKIQDSLSSTASALGKLQDVVNHNAQA
OL677199.1/BA.1/Omicron    AMQMAYRFNGIGVTQNVLYENQKLIANQFNSAIGKIQDSLSSTASALGKLQDVVNHNAQA
OM993050.1/BA.1/Omicron    AMQMAYRFNGIGVTQNVLYENQKLIANQFNSAIGKIQDSLSSTASALGKLQDVVNHNAQA
ON003903.1/BA.1/Omicron    AMQMAYRFNGIGVTQNVLYENQKLIANQFNSAIGKIQDSLSSTASALGKLQDVVNHNAQA
ON051766.1/BA.1/Omicron    AMQMAYRFNGIGVTQNVLYENQKLIANQFNSAIGKIQDSLSSTASALGKLQDVVNHNAQA
ON051889.1/BA.1/Omicron    AMQMAYRFNGIGVTQNVLYENQKLIANQFNSAIGKIQDSLSSTASALGKLQDVVNHNAQA
ON071741.1/BA.1/Omicron    AMQMAYRFNGIGVTQNVLYENQKLIANQFNSAIGKIQDSLSSTASALGKLQDVVNHNAQA
ON071466.1/BA.1/Omicron    AMQMAYRFNGIGVTQNVLYENQKLIANQFNSAIGKIQDSLSSTASALGKLQDVVNHNAQA
OP315455.1/BA.1/Omicron    AMQMAYRFNGIGVTQNVLYENQKLIANQFNSAIGKIQDSLSSTASALGKLQDVVNHNAQA
ON071518.1/BA.1/Omicron    AMQMAYRFNGIGVTQNVLYENQKLIANQFNSAIGKIQDSLSSTASALGKLQDVVNHNAQA
ON772104.1/BA.5.2/Omicron  AMQMAYRFNGIGVTQNVLYENQKLIANQFNSAIGKIQDSLSSTASALGKLQDVVNHNAQA
ON867566.1/BA.5.2/Omicron  AMQMAYRFNGIGVTQNVLYENQKLIANQFNSAIGKIQDSLSSTASALGKLQDVVNHNAQA
ON861467.1/BA.5.2/Omicron  AMQMAYRFNGIGVTQNVLYENQKLIANQFNSAIGKIQDSLSSTASALGKLQDVVNHNAQA
OP159825.1/BA.5.2/Omicron  AMQMAYRFNGIGVTQNVLYENQKLIANQFNSAIGKIQDSLSSTASALGKLQDVVNHNAQA
OP312522.1/BA.5.2/Omicron  AMQMAYRFNGIGVTQNVLYENQKLIANQFNSAIGKIQDSLSSTASALGKLQDVVNHNAQA
OP401897.1/BA.5.2/Omicron  AMQMAYRFNGIGVTQNVLYENQKLIANQFNSAIGKIQDSLSSTASALGKLQDVVNHNAQA
OP406330.1/BA.5.2/Omicron  AMQMAYRFNGIGVTQNVLYENQKLIANQFNSAIGKIQDSLSSTASALGKLQDVVNHNAQA
OP596593.1/BA.5.2/Omicron  AMQMAYRFNGIGVTQNVLYENQKLIANQFNSAIGKIQDSLSSTASALGKLQDVVNHNAQA
OP660471.1/BA.5.2/Omicron  AMQMAYRFNGIGVTQNVLYENQKLIANQFNSAIGKIQDSLSSTASALGKLQDVVNHNAQA
OP660514.1/BA.5.2/Omicron  AMQMAYRFNGIGVTQNVLYENQKLIANQFNSAIGKIQDSLSSTASALGKLQDVVNHNAQA
OP619697.1/BF.7/Omicron    AMQMAYRFNGIGVTQNVLYENQKLIANQFNSAIGKIQDSLSSTASALGKLQDVVNHNAQA
OP624633.1/BF.7/Omicron    AMQMAYRFNGIGVTQNVLYENQKLIANQFNSAIGKIQDSLSSTASALGKLQDVVNHNAQA
OP625651.1/BF.7/Omicron    AMQMAYRFNGIGVTQNVLYENQKLIANQFNSAIGKIQDSLSSTASALGKLQDVVNHNAQA
OP649145.1/BF.7/Omicron    AMQMAYRFNGIGVTQNVLYENQKLIANQFNSAIGKIQDSLSSTASALGKLQDVVNHNAQA
OP651402.1/BF.7/Omicron    AMQMAYRFNGIGVTQNVLYENQKLIANQFNSAIGKIQDSLSSTASALGKLQDVVNHNAQA
OP652819.1/BF.7/Omicron    AMQMAYRFNGIGVTQNVLYENQKLIANQFNSAIGKIQDSLSSTASALGKLQDVVNHNAQA
OP707921.1/BF.7/Omicron    AMQMAYRFNGIGVTQNVLYENQKLIANQFNSAIGKIQDSLSSTASALGKLQDVVNHNAQA
OP754604.1/BF.7/Omicron    AMQMAYRFNGIGVTQNVLYENQKLIANQFNSAIGKIQDSLSSTASALGKLQDVVNHNAQA
OQ778124.1/BF.7/Omicron    AMQMAYRFNGIGVTQNVLYENQKLIANQFNSAIGKIQDSLSSTASALGKLQDVVNHNAQA
OQ792514.1/BF.7/Omicron    AMQMAYRFNGIGVTQNVLYENQKLIANQFNSAIGKIQDSLSSTASALGKLQDVVNHNAQA
```

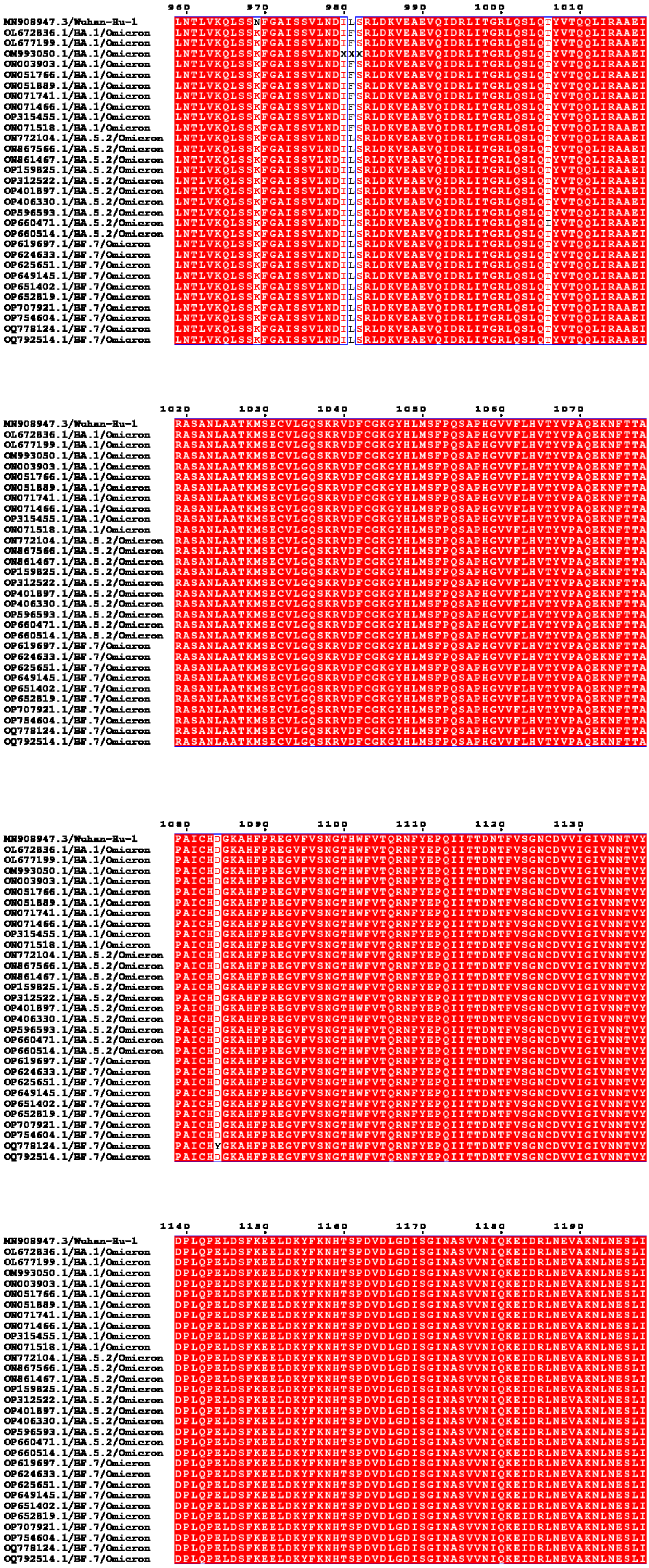

960 970 980 990 1000 1010
MN908947.3/Wuhan-Hu-1 LNTLVKQLSSNFGAISSVLNDILSRLDKVEAEVQIDRLITGRLQSLQTYVTQQLIRAAEI
OL672836.1/BA.1/Omicron LNTLVKQLSSKFGAISSVLNDIFSRLDKVEAEVQIDRLITGRLQSLQTYVTQQLIRAAEI
OL677199.1/BA.1/Omicron LNTLVKQLSSKFGAISSVLNDIFSRLDKVEAEVQIDRLITGRLQSLQTYVTQQLIRAAEI
OM993050.1/BA.1/Omicron LNTLVKQLSSKFGAISSVLNDXXXRLDKVEAEVQIDRLITGRLQSLQTYVTQQLIRAAEI
ON003903.1/BA.1/Omicron LNTLVKQLSSKFGAISSVLNDIFSRLDKVEAEVQIDRLITGRLQSLQTYVTQQLIRAAEI
ON051766.1/BA.1/Omicron LNTLVKQLSSKFGAISSVLNDIFSRLDKVEAEVQIDRLITGRLQSLQTYVTQQLIRAAEI
ON051889.1/BA.1/Omicron LNTLVKQLSSKFGAISSVLNDIFSRLDKVEAEVQIDRLITGRLQSLQTYVTQQLIRAAEI
ON071741.1/BA.1/Omicron LNTLVKQLSSKFGAISSVLNDIFSRLDKVEAEVQIDRLITGRLQSLQTYVTQQLIRAAEI
ON071466.1/BA.1/Omicron LNTLVKQLSSKFGAISSVLNDIFSRLDKVEAEVQIDRLITGRLQSLQTYVTQQLIRAAEI
OP315455.1/BA.1/Omicron LNTLVKQLSSKFGAISSVLNDIFSRLDKVEAEVQIDRLITGRLQSLQTYVTQQLIRAAEI
ON071518.1/BA.1/Omicron LNTLVKQLSSKFGAISSVLNDIFSRLDKVEAEVQIDRLITGRLQSLQTYVTQQLIRAAEI
ON772104.1/BA.5.2/Omicron LNTLVKQLSSKFGAISSVLNDILSRLDKVEAEVQIDRLITGRLQSLQTYVTQQLIRAAEI
ON867566.1/BA.5.2/Omicron LNTLVKQLSSKFGAISSVLNDILSRLDKVEAEVQIDRLITGRLQSLQTYVTQQLIRAAEI
ON861467.1/BA.5.2/Omicron LNTLVKQLSSKFGAISSVLNDILSRLDKVEAEVQIDRLITGRLQSLQTYVTQQLIRAAEI
OP159825.1/BA.5.2/Omicron LNTLVKQLSSKFGAISSVLNDILSRLDKVEAEVQIDRLITGRLQSLQTYVTQQLIRAAEI
OP312522.1/BA.5.2/Omicron LNTLVKQLSSKFGAISSVLNDILSRLDKVEAEVQIDRLITGRLQSLQTYVTQQLIRAAEI
OP401897.1/BA.5.2/Omicron LNTLVKQLSSKFGAISSVLNDILSRLDKVEAEVQIDRLITGRLQSLQTYVTQQLIRAAEI
OP406330.1/BA.5.2/Omicron LNTLVKQLSSKFGAISSVLNDILSRLDKVEAEVQIDRLITGRLQSLQTYVTQQLIRAAEI
OP596593.1/BA.5.2/Omicron LNTLVKQLSSKFGAISSVLNDILSRLDKVEAEVQIDRLITGRLQSLQTYVTQQLIRAAEI
OP660471.1/BA.5.2/Omicron LNTLVKQLSSKFGAISSVLNDILSRLDKVEAEVQIDRLITGRLQSLQIYVTQQLIRAAEI
OP660514.1/BA.5.2/Omicron LNTLVKQLSSKFGAISSVLNDILSRLDKVEAEVQIDRLITGRLQSLQTYVTQQLIRAAEI
OP619697.1/BF.7/Omicron LNTLVKQLSSKFGAISSVLNDILSRLDKVEAEVQIDRLITGRLQSLQTYVTQQLIRAAEI
OP624633.1/BF.7/Omicron LNTLVKQLSSKFGAISSVLNDILSRLDKVEAEVQIDRLITGRLQSLQTYVTQQLIRAAEI
OP625651.1/BF.7/Omicron LNTLVKQLSSKFGAISSVLNDILSRLDKVEAEVQIDRLITGRLQSLQTYVTQQLIRAAEI
OP649145.1/BF.7/Omicron LNTLVKQLSSKFGAISSVLNDILSRLDKVEAEVQIDRLITGRLQSLQTYVTQQLIRAAEI
OP651402.1/BF.7/Omicron LNTLVKQLSSKFGAISSVLNDILSRLDKVEAEVQIDRLITGRLQSLQTYVTQQLIRAAEI
OP652819.1/BF.7/Omicron LNTLVKQLSSKFGAISSVLNDILSRLDKVEAEVQIDRLITGRLQSLQTYVTQQLIRAAEI
OP707921.1/BF.7/Omicron LNTLVKQLSSKFGAISSVLNDILSRLDKVEAEVQIDRLITGRLQSLQTYVTQQLIRAAEI
OP754604.1/BF.7/Omicron LNTLVKQLSSKFGAISSVLNDILSRLDKVEAEVQIDRLITGRLQSLQTYVTQQLIRAAEI
OQ778124.1/BF.7/Omicron LNTLVKQLSSKFGAISSVLNDILSRLDKVEAEVQIDRLITGRLQSLQTYVTQQLIRAAEI
OQ792514.1/BF.7/Omicron LNTLVKQLSSKFGAISSVLNDILSRLDKVEAEVQIDRLITGRLQSLQTYVTQQLIRAAEI
1020 1030 1040 1050 1060 1070
MN908947.3/Wuhan-Hu-1 RASANLAATKMSECVLGQSKRVDFCGKGYHLMSFPQSAPHGVVFLHVTYVPAQEKNFTTA
OL672836.1/BA.1/Omicron RASANLAATKMSECVLGQSKRVDFCGKGYHLMSFPQSAPHGVVFLHVTYVPAQEKNFTTA
OL677199.1/BA.1/Omicron RASANLAATKMSECVLGQSKRVDFCGKGYHLMSFPQSAPHGVVFLHVTYVPAQEKNFTTA
OM993050.1/BA.1/Omicron RASANLAATKMSECVLGQSKRVDFCGKGYHLMSFPQSAPHGVVFLHVTYVPAQEKNFTTA
ON003903.1/BA.1/Omicron RASANLAATKMSECVLGQSKRVDFCGKGYHLMSFPQSAPHGVVFLHVTYVPAQEKNFTTA
ON051766.1/BA.1/Omicron RASANLAATKMSECVLGQSKRVDFCGKGYHLMSFPQSAPHGVVFLHVTYVPAQEKNFTTA
ON051889.1/BA.1/Omicron RASANLAATKMSECVLGQSKRVDFCGKGYHLMSFPQSAPHGVVFLHVTYVPAQEKNFTTA
ON071741.1/BA.1/Omicron RASANLAATKMSECVLGQSKRVDFCGKGYHLMSFPQSAPHGVVFLHVTYVPAQEKNFTTA
ON071466.1/BA.1/Omicron RASANLAATKMSECVLGQSKRVDFCGKGYHLMSFPQSAPHGVVFLHVTYVPAQEKNFTTA
OP315455.1/BA.1/Omicron RASANLAATKMSECVLGQSKRVDFCGKGYHLMSFPQSAPHGVVFLHVTYVPAQEKNFTTA
ON071518.1/BA.1/Omicron RASANLAATKMSECVLGQSKRVDFCGKGYHLMSFPQSAPHGVVFLHVTYVPAQEKNFTTA
ON772104.1/BA.5.2/Omicron RASANLAATKMSECVLGQSKRVDFCGKGYHLMSFPQSAPHGVVFLHVTYVPAQEKNFTTA
ON867566.1/BA.5.2/Omicron RASANLAATKMSECVLGQSKRVDFCGKGYHLMSFPQSAPHGVVFLHVTYVPAQEKNFTTA
ON861467.1/BA.5.2/Omicron RASANLAATKMSECVLGQSKRVDFCGKGYHLMSFPQSAPHGVVFLHVTYVPAQEKNFTTA
OP159825.1/BA.5.2/Omicron RASANLAATKMSECVLGQSKRVDFCGKGYHLMSFPQSAPHGVVFLHVTYVPAQEKNFTTA
OP312522.1/BA.5.2/Omicron RASANLAATKMSECVLGQSKRVDFCGKGYHLMSFPQSAPHGVVFLHVTYVPAQEKNFTTA
OP401897.1/BA.5.2/Omicron RASANLAATKMSECVLGQSKRVDFCGKGYHLMSFPQSAPHGVVFLHVTYVPAQEKNFTTA
OP406330.1/BA.5.2/Omicron RASANLAATKMSECVLGQSKRVDFCGKGYHLMSFPQSAPHGVVFLHVTYVPAQEKNFTTA
OP596593.1/BA.5.2/Omicron RASANLAATKMSECVLGQSKRVDFCGKGYHLMSFPQSAPHGVVFLHVTYVPAQEKNFTTA
OP660471.1/BA.5.2/Omicron RASANLAATKMSECVLGQSKRVDFCGKGYHLMSFPQSAPHGVVFLHVTYVPAQEKNFTTA
OP660514.1/BA.5.2/Omicron RASANLAATKMSECVLGQSKRVDFCGKGYHLMSFPQSAPHGVVFLHVTYVPAQEKNFTTA
OP619697.1/BF.7/Omicron RASANLAATKMSECVLGQSKRVDFCGKGYHLMSFPQSAPHGVVFLHVTYVPAQEKNFTTA
OP624633.1/BF.7/Omicron RASANLAATKMSECVLGQSKRVDFCGKGYHLMSFPQSAPHGVVFLHVTYVPAQEKNFTTA
OP625651.1/BF.7/Omicron RASANLAATKMSECVLGQSKRVDFCGKGYHLMSFPQSAPHGVVFLHVTYVPAQEKNFTTA
OP649145.1/BF.7/Omicron RASANLAATKMSECVLGQSKRVDFCGKGYHLMSFPQSAPHGVVFLHVTYVPAQEKNFTTA
OP651402.1/BF.7/Omicron RASANLAATKMSECVLGQSKRVDFCGKGYHLMSFPQSAPHGVVFLHVTYVPAQEKNFTTA
OP652819.1/BF.7/Omicron RASANLAATKMSECVLGQSKRVDFCGKGYHLMSFPQSAPHGVVFLHVTYVPAQEKNFTTA
OP707921.1/BF.7/Omicron RASANLAATKMSECVLGQSKRVDFCGKGYHLMSFPQSAPHGVVFLHVTYVPAQEKNFTTA
OP754604.1/BF.7/Omicron RASANLAATKMSECVLGQSKRVDFCGKGYHLMSFPQSAPHGVVFLHVTYVPAQEKNFTTA
OQ778124.1/BF.7/Omicron RASANLAATKMSECVLGQSKRVDFCGKGYHLMSFPQSAPHGVVFLHVTYVPAQEKNFTTA
OQ792514.1/BF.7/Omicron RASANLAATKMSECVLGQSKRVDFCGKGYHLMSFPQSAPHGVVFLHVTYVPAQEKNFTTA
1080 1090 1100 1110 1120 1130
MN908947.3/Wuhan-Hu-1 PAICHDGKAHFPREGVFVSNGTHWFVTQRNFYEPQIITTDNTFVSGNCDVVIGIVNNTVY
OL672836.1/BA.1/Omicron PAICHDGKAHFPREGVFVSNGTHWFVTQRNFYEPQIITTDNTFVSGNCDVVIGIVNNTVY
OL677199.1/BA.1/Omicron PAICHDGKAHFPREGVFVSNGTHWFVTQRNFYEPQIITTDNTFVSGNCDVVIGIVNNTVY
OM993050.1/BA.1/Omicron PAICHDGKAHFPREGVFVSNGTHWFVTQRNFYEPQIITTDNTFVSGNCDVVIGIVNNTVY
ON003903.1/BA.1/Omicron PAICHDGKAHFPREGVFVSNGTHWFVTQRNFYEPQIITTDNTFVSGNCDVVIGIVNNTVY
ON051766.1/BA.1/Omicron PAICHDGKAHFPREGVFVSNGTHWFVTQRNFYEPQIITTDNTFVSGNCDVVIGIVNNTVY
ON051889.1/BA.1/Omicron PAICHDGKAHFPREGVFVSNGTHWFVTQRNFYEPQIITTDNTFVSGNCDVVIGIVNNTVY
ON071741.1/BA.1/Omicron PAICHDGKAHFPREGVFVSNGTHWFVTQRNFYEPQIITTDNTFVSGNCDVVIGIVNNTVY
ON071466.1/BA.1/Omicron PAICHDGKAHFPREGVFVSNGTHWFVTQRNFYEPQIITTDNTFVSGNCDVVIGIVNNTVY
OP315455.1/BA.1/Omicron PAICHDGKAHFPREGVFVSNGTHWFVTQRNFYEPQIITTDNTFVSGNCDVVIGIVNNTVY
ON071518.1/BA.1/Omicron PAICHDGKAHFPREGVFVSNGTHWFVTQRNFYEPQIITTDNTFVSGNCDVVIGIVNNTVY
ON772104.1/BA.5.2/Omicron PAICHDGKAHFPREGVFVSNGTHWFVTQRNFYEPQIITTDNTFVSGNCDVVIGIVNNTVY
ON867566.1/BA.5.2/Omicron PAICHDGKAHFPREGVFVSNGTHWFVTQRNFYEPQIITTDNTFVSGNCDVVIGIVNNTVY
ON861467.1/BA.5.2/Omicron PAICHDGKAHFPREGVFVSNGTHWFVTQRNFYEPQIITTDNTFVSGNCDVVIGIVNNTVY
OP159825.1/BA.5.2/Omicron PAICHDGKAHFPREGVFVSNGTHWFVTQRNFYEPQIITTDNTFVSGNCDVVIGIVNNTVY
OP312522.1/BA.5.2/Omicron PAICHDGKAHFPREGVFVSNGTHWFVTQRNFYEPQIITTDNTFVSGNCDVVIGIVNNTVY
OP401897.1/BA.5.2/Omicron PAICHDGKAHFPREGVFVSNGTHWFVTQRNFYEPQIITTDNTFVSGNCDVVIGIVNNTVY
OP406330.1/BA.5.2/Omicron PAICHDGKAHFPREGVFVSNGTHWFVTQRNFYEPQIITTDNTFVSGNCDVVIGIVNNTVY
OP596593.1/BA.5.2/Omicron PAICHDGKAHFPREGVFVSNGTHWFVTQRNFYEPQIITTDNTFVSGNCDVVIGIVNNTVY
OP660471.1/BA.5.2/Omicron PAICHDGKAHFPREGVFVSNGTHWFVTQRNFYEPQIITTDNTFVSGNCDVVIGIVNNTVY
OP660514.1/BA.5.2/Omicron PAICHDGKAHFPREGVFVSNGTHWFVTQRNFYEPQIITTDNTFVSGNCDVVIGIVNNTVY
OP619697.1/BF.7/Omicron PAICHDGKAHFPREGVFVSNGTHWFVTQRNFYEPQIITTDNTFVSGNCDVVIGIVNNTVY
OP624633.1/BF.7/Omicron PAICHDGKAHFPREGVFVSNGTHWFVTQRNFYEPQIITTDNTFVSGNCDVVIGIVNNTVY
OP625651.1/BF.7/Omicron PAICHDGKAHFPREGVFVSNGTHWFVTQRNFYEPQIITTDNTFVSGNCDVVIGIVNNTVY
OP649145.1/BF.7/Omicron PAICHDGKAHFPREGVFVSNGTHWFVTQRNFYEPQIITTDNTFVSGNCDVVIGIVNNTVY
OP651402.1/BF.7/Omicron PAICHDGKAHFPREGVFVSNGTHWFVTQRNFYEPQIITTDNTFVSGNCDVVIGIVNNTVY
OP652819.1/BF.7/Omicron PAICHDGKAHFPREGVFVSNGTHWFVTQRNFYEPQIITTDNTFVSGNCDVVIGIVNNTVY
OP707921.1/BF.7/Omicron PAICHDGKAHFPREGVFVSNGTHWFVTQRNFYEPQIITTDNTFVSGNCDVVIGIVNNTVY
OP754604.1/BF.7/Omicron PAICHDGKAHFPREGVFVSNGTHWFVTQRNFYEPQIITTDNTFVSGNCDVVIGIVNNTVY
OQ778124.1/BF.7/Omicron PAICHYGKAHFPREGVFVSNGTHWFVTQRNFYEPQIITTDNTFVSGNCDVVIGIVNNTVY
OQ792514.1/BF.7/Omicron PAICHDGKAHFPREGVFVSNGTHWFVTQRNFYEPQIITTDNTFVSGNCDVVIGIVNNTVY
1140 1150 1160 1170 1180 1190
MN908947.3/Wuhan-Hu-1 DPLQPELDSFKEELDKYFKNHTSPDVDLGDISGINASVVNIQKEIDRLNEVAKNLNESLI
OL672836.1/BA.1/Omicron DPLQPELDSFKEELDKYFKNHTSPDVDLGDISGINASVVNIQKEIDRLNEVAKNLNESLI
OL677199.1/BA.1/Omicron DPLQPELDSFKEELDKYFKNHTSPDVDLGDISGINASVVNIQKEIDRLNEVAKNLNESLI
OM993050.1/BA.1/Omicron DPLQPELDSFKEELDKYFKNHTSPDVDLGDISGINASVVNIQKEIDRLNEVAKNLNESLI
ON003903.1/BA.1/Omicron DPLQPELDSFKEELDKYFKNHTSPDVDLGDISGINASVVNIQKEIDRLNEVAKNLNESLI
ON051766.1/BA.1/Omicron DPLQPELDSFKEELDKYFKNHTSPDVDLGDISGINASVVNIQKEIDRLNEVAKNLNESLI
ON051889.1/BA.1/Omicron DPLQPELDSFKEELDKYFKNHTSPDVDLGDISGINASVVNIQKEIDRLNEVAKNLNESLI
ON071741.1/BA.1/Omicron DPLQPELDSFKEELDKYFKNHTSPDVDLGDISGINASVVNIQKEIDRLNEVAKNLNESLI
ON071466.1/BA.1/Omicron DPLQPELDSFKEELDKYFKNHTSPDVDLGDISGINASVVNIQKEIDRLNEVAKNLNESLI
OP315455.1/BA.1/Omicron DPLQPELDSFKEELDKYFKNHTSPDVDLGDISGINASVVNIQKEIDRLNEVAKNLNESLI
ON071518.1/BA.1/Omicron DPLQPELDSFKEELDKYFKNHTSPDVDLGDISGINASVVNIQKEIDRLNEVAKNLNESLI
ON772104.1/BA.5.2/Omicron DPLQPELDSFKEELDKYFKNHTSPDVDLGDISGINASVVNIQKEIDRLNEVAKNLNESLI
ON867566.1/BA.5.2/Omicron DPLQPELDSFKEELDKYFKNHTSPDVDLGDISGINASVVNIQKEIDRLNEVAKNLNESLI
ON861467.1/BA.5.2/Omicron DPLQPELDSFKEELDKYFKNHTSPDVDLGDISGINASVVNIQKEIDRLNEVAKNLNESLI
OP159825.1/BA.5.2/Omicron DPLQPELDSFKEELDKYFKNHTSPDVDLGDISGINASVVNIQKEIDRLNEVAKNLNESLI
OP312522.1/BA.5.2/Omicron DPLQPELDSFKEELDKYFKNHTSPDVDLGDISGINASVVNIQKEIDRLNEVAKNLNESLI
OP401897.1/BA.5.2/Omicron DPLQPELDSFKEELDKYFKNHTSPDVDLGDISGINASVVNIQKEIDRLNEVAKNLNESLI
OP406330.1/BA.5.2/Omicron DPLQPELDSFKEELDKYFKNHTSPDVDLGDISGINASVVNIQKEIDRLNEVAKNLNESLI
OP596593.1/BA.5.2/Omicron DPLQPELDSFKEELDKYFKNHTSPDVDLGDISGINASVVNIQKEIDRLNEVAKNLNESLI
OP660471.1/BA.5.2/Omicron DPLQPELDSFKEELDKYFKNHTSPDVDLGDISGINASVVNIQKEIDRLNEVAKNLNESLI
OP660514.1/BA.5.2/Omicron DPLQPELDSFKEELDKYFKNHTSPDVDLGDISGINASVVNIQKEIDRLNEVAKNLNESLI
OP619697.1/BF.7/Omicron DPLQPELDSFKEELDKYFKNHTSPDVDLGDISGINASVVNIQKEIDRLNEVAKNLNESLI
OP624633.1/BF.7/Omicron DPLQPELDSFKEELDKYFKNHTSPDVDLGDISGINASVVNIQKEIDRLNEVAKNLNESLI
OP625651.1/BF.7/Omicron DPLQPELDSFKEELDKYFKNHTSPDVDLGDISGINASVVNIQKEIDRLNEVAKNLNESLI
OP649145.1/BF.7/Omicron DPLQPELDSFKEELDKYFKNHTSPDVDLGDISGINASVVNIQKEIDRLNEVAKNLNESLI
OP651402.1/BF.7/Omicron DPLQPELDSFKEELDKYFKNHTSPDVDLGDISGINASVVNIQKEIDRLNEVAKNLNESLI
OP652819.1/BF.7/Omicron DPLQPELDSFKEELDKYFKNHTSPDVDLGDISGINASVVNIQKEIDRLNEVAKNLNESLI
OP707921.1/BF.7/Omicron DPLQPELDSFKEELDKYFKNHTSPDVDLGDISGINASVVNIQKEIDRLNEVAKNLNESLI
OP754604.1/BF.7/Omicron DPLQPELDSFKEELDKYFKNHTSPDVDLGDISGINASVVNIQKEIDRLNEVAKNLNESLI
OQ778124.1/BF.7/Omicron DPLQPELDSFKEELDKYFKNHTSPDVDLGDISGINASVVNIQKEIDRLNEVAKNLNESLI
OQ792514.1/BF.7/Omicron DPLQPELDSFKEELDKYFKNHTSPDVDLGDISGINASVVNIQKEIDRLNEVAKNLNESLI

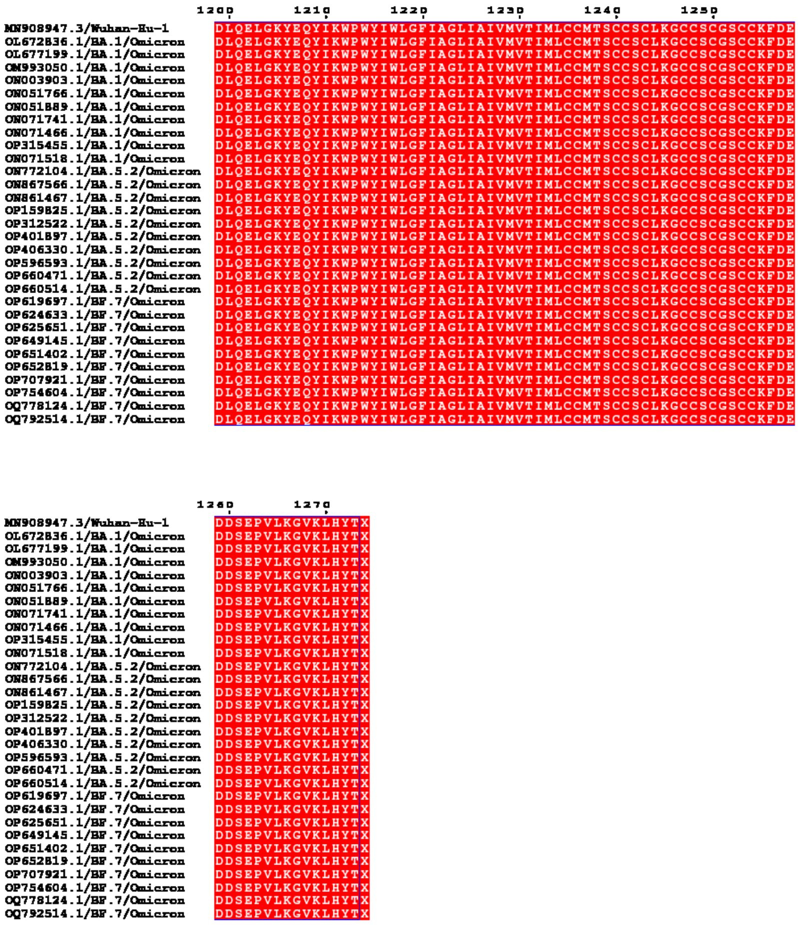

Figure S2. Multiple alignment of amino acid sequences of spike protein from Omicron sub-lineages BA.1, BA.5.2 and BF.7 collected from different regions, and compared with wild-type SARS-CoV-2 strain isolated from Wuhan, China (accession number MN908947). The accession number and pangolin lineage are shown on the left side of the sequences. The red boxes represent identical residues while similar residues are indicated by red letters.
